# Front line defenders of the ecological niche! Screening the structural diversity of peptaibiotics from saprotrophic and fungicolous *Trichoderma/Hypocrea* species

**DOI:** 10.1007/s13225-013-0276-z

**Published:** 2014-11-01

**Authors:** Christian R. Röhrich, Walter M. Jaklitsch, Hermann Voglmayr, Anita Iversen, Andreas Vilcinskas, Kristian Fog Nielsen, Ulf Thrane, Hans von Döhren, Hans Brückner, Thomas Degenkolb

**Affiliations:** Bioresources Project Group, Fraunhofer Institute for Molecular Biology and Applied Ecology (IME), Winchesterstrasse 2, 35394 Giessen, Germany. *Present Address:* AB SCIEX Germany GmbH, Landwehrstrasse 54, 64293 Darmstadt, Germany; Department of Systematic and Evolutionary Botany, Faculty Centre of Biodiversity, University of Vienna, Rennweg 14, 1030 Vienna, Austria; Department of Systematic and Evolutionary Botany, Faculty Centre of Biodiversity, University of Vienna, Rennweg 14, 1030 Vienna, Austria; Department of Systems Biology, Technical University of Denmark, Søltofts Plads, Building 221, 2800 Kgs. Lyngby, Denmark. *Present Address:*Danish Emergency Management Agency, Universitetsparken 2, 2100 Copenhagen, Denmark; Bioresources Project Group, Fraunhofer Institute for Molecular Biology and Applied Ecology (IME), Winchesterstrasse 2, 35394 Giessen, Germany; Interdisciplinary Research Centre for BioSystems, Land Use and Nutrition (IFZ), Department of Applied Entomology, Institute of Phytopathology and Applied Zoology (IPAZ), University of Giessen, Heinrich-Buff-Ring 26-32, 35392 Giessen, Germany; Department of Systems Biology, Technical University of Denmark, Søltofts Plads, Building 221, 2800 Kgs. Lyngby, Denmark; Department of Systems Biology, Technical University of Denmark, Søltofts Plads, Building 221, 2800 Kgs. Lyngby, Denmark; Biochemistry and Molecular Biology OE 2, Institute of Chemistry, Technical University of Berlin, Franklinstrasse 29, 10587 Berlin, Germany; Interdisciplinary Research Centre for BioSystems, Land Use and Nutrition (IFZ), Department of Food Sciences, Institute of Nutritional Science, University of Giessen, Heinrich-Buff-Ring 26-32, 35392 Giessen, Germany; Interdisciplinary Research Centre for BioSystems, Land Use and Nutrition (IFZ), Department of Applied Entomology, Institute of Phytopathology and Applied Zoology (IPAZ), University of Giessen, Heinrich-Buff-Ring 26-32, 35392 Giessen, Germany thomas.degenkolb@ernaehrung.uni-giessen.de

**Keywords:** HPLC/QTOF-ESI-HRMS, Metabolite profiling, Peptaibiotics, Peptaibols, Aib peptides, *Trichoderma*, *Hypocrea*

## Abstract

Approximately 950 individual sequences of non-ribosomally biosynthesised peptides are produced by the genus *Trichoderma*/*Hypocrea* that belong to a perpetually growing class of mostly linear antibiotic oligopeptides, which are rich in the non-proteinogenic *α*-aminoisobutyric acid (Aib). Thus, they are comprehensively named peptaibiotics. Notably, peptaibiotics represent ca. 80 % of the total inventory of secondary metabolites currently known from *Trichoderma/Hypocrea*. Their unique membrane-modifying bioactivity results from amphipathicity and helicity, thus making them ideal candidates in assisting both colonisation and defence of the natural habitats by their fungal producers. Despite this, reports on the in vivo-detection of peptaibiotics have scarcely been published in the past. In order to evaluate the significance of peptaibiotic production for a broader range of potential producers, we screened nine specimens belonging to seven hitherto uninvestigated fungicolous or saprotrophic *Trichoderma/Hypocrea* species by liquid chromatography coupled to electrospray high resolution mass spectrometry. Sequences of peptaibiotics found were independently confirmed by analysing the peptaibiome of pure agar cultures obtained by single-ascospore isolation from the specimens. Of the nine species examined, five were screened positive for peptaibiotics. A total of 78 peptaibiotics were sequenced, 56 (=72 %) of which are new. Notably, dihydroxyphenylalaninol and *O*-prenylated tyrosinol, two *C*-terminal residues, which have not been reported for peptaibiotics before, were found as well as new and recurrent sequences carrying the recently described tyrosinol residue at their *C*-terminus. The majority of peptaibiotics sequenced are 18- or 19-residue peptaibols. Structural homologies with ‘classical representatives’ of subfamily 1 (SF1)-peptaibiotics argue for the formation of transmembrane ion channels, which are prone to facilitate the producer capture and defence of its substratum.

## Introduction

Currently, the fungal genus *Trichoderma/Hypocrea*^1^ comprises more than 200 validly described species, which have been recognised by molecular phylogenetic analysis (Atanasova et al. 2013). This high taxonomic diversity in *Trichoderma/Hypocrea* is not only reflected in a permanently increasing number of species (Jaklitsch 2009, 2011; Jaklitsch and Voglmayr 2012; Jaklitsch et al. 2012, 2013; Chaverri et al. 2011; Samuels and Ismaiel 2011, Samuels et al. 2012a,b; Kim et al. 2012, 2013; Yamaguchi et al. 2012; Li et al. 2013; López-Quintero et al. 2013, Yabuki et al. 2014), but also in a fast-growing number of secondary metabolites of remarkable structural diversity. The latter include low-molecular-weight compounds such as pyrones (Jeleń et al. 2013), butenolides, terpenes, and steroids, but also *N*-heterocyclic compounds and isocyanides. In addition to these relatively nonpolar and often partly volatile compounds, an impressive inventory of non-volatile compounds, comprising some alkaloids and an imposing number of peptide antibiotics, is produced. Reino et al. (2008) reviewed 186 compounds; however, peptaibiotics (see below) were treated only marginally and incomprehensively. As of August 2013, a total of 501 entries are recorded for *Trichoderma* (461) and *Hypocrea* (40) in AntiBase, more than 300 of which are N-containing, including less than 100 in the range of 50–800 Da (Laatsch 2013).

Considering recent publications in this field, which have not yet been included into AntiBase 2013 (Table 1), an estimate of 225 to 250 non-peptaibiotic secondary metabolites from *Trichoderma/Hypocrea* seems appropriate. However, the overwhelming majority of secondary metabolites obtained from this genus so far belong to a perpetually growing family of non-ribosomally biosynthesised, linear or, in a few cases, cyclic peptide antibiotics of exclusively fungal origin, comprehensively named peptaibiotics:

According to the definition, the members of this peptide family show, besides proteinogenic amino acids, *i*) a relatively high content of the marker *α*-aminoisobutyric acid (Aib), which is often accompanied by other *α*,*α*-dialkyl *α*-amino acids such as D- and/or L-isovaline (Iva) or, occasionally, *α*-ethylnorvaline (EtNva), or 1-aminocyclopropane-1-carboxylic acid (Acc); *ii*) have a molecular weight between 500 and 2,100 Da, thus containing 4–21 residues; *iii*) are characterised by the presence of other non-proteinogenic amino acids and/or lipoamino acids; *iv*) possess an acylated *N*-terminus, and *v*) in the case of linear peptides, have a *C*-terminal residue that most frequently consists of an amide-bonded *β*-amino alcohol, thus defining the largest subfamily of peptaibiotics, named peptaibols. Alternatively, the *C*-terminus might also be a polyamine, amide, free amino acid, 2,5-diketopiperazine, or a sugar alcohol (Degenkolb and Brückner 2008; Stoppacher et al. 2013).

Of the approximately 1,250 to 1,300 individual sequences of peptaibiotics known as of autumn 2013 (Ayers et al. 2012; Carroux et al. 2013; Figueroa et al. 2013; Kimonyo and Brückner 2013; Röhrich et al. 2012; Röhrich et al. 2013a, b; Chen et al. 2013; Panizel et al. 2013; Ren et al. 2013; Stoppacher et al. 2013), about 950 have been obtained from *Trichoderma/Hypocrea* species, thus confirming the genus as the most prolific source of this group of non-ribosomal peptide antibiotics (Brückner et al. 1991; Degenkolb and Brückner 2008; Brückner et al. 2009).

Both the taxonomic and metabolic diversity of *Trichoderma/Hypocrea* are hypothesised to originate from mycoparasitism or hyperparasitism, which may represent the ancestral life style of this genus (Kubicek et al. 2011). The unique bioactivities of peptaibiotics, resulting from their amphipathicity and helicity, make them ideal candidates to support the parasitic life style of their fungal producers:

Under in vitro-conditions, the parallel formation of peptaibiotics such as the 19-residue trichorzianins^2^ and of hydrolytic enzymes, above all chitinases and *β*-1,3-glucanases (Schirmböck et al. 1994), could be demonstrated. This observation led to a widely accepted model describing the synergistic interaction of peptaibiotics and hydrolases in the course of mycoparasitism of *Trichoderma atroviride* towards *Botrytis cinerea* (Lorito et al. 1996). Despite this, reports on in vivo-detection of peptaibiotics have scarcely been published in the past. Examples include the isolation of hypelcins A and B obtained from ca. 2 kg of dried, crushed stromata of the mycoparasite *Hypocrea peltata* (Fujita et al. 1984; Matsuura et al. 1993, 1994)^3^ as well as the detection of antiamoebins in herbivore dung, which have been produced by the coprophilous *Stilbella fimetaria* (syn. *S. erythrocephala*) (Lehr et al. 2006).

In order to close this gap, we initiated a screening project aimed at resolving the question as to whether peptaibiotic production in vivo is a common adaptation strategy of *Trichoderma/Hypocrea* species for colonising and defending ecological niches:

Several *Hypocrea* specimens were freshly collected in the natural habitat and analysed for the presence of peptaibiotics. Sequences of peptaibiotics found were independently confirmed by analysing the peptaibiome^4^ of pure agar cultures obtained by single-ascospore isolation from the specimens. Using liquid chromatography coupled to electrospray high resolution mass spectrometry we succeeded in detecting 28 peptaibiotics from the polyporicolous *Hypocrea pulvinata* (Röhrich et al. 2012). Another 49 peptaibiotics were sequenced in *Hypocrea phellinicola*, a parasite of *Phellinus* sp., especially *Ph. ferruginosus* (Röhrich et al. 2013a).

Due to these encouraging results, our screening programme was extended to another nine specimens belonging to seven hitherto uninvestigated mycoparasitic or saprotrophic *Trichoderma/Hypocrea* species, respectively (Table 2).

## Materials and methods

Specimens of *Hypocrea* teleomorphs were collected from four different locations in Austria (Table 3). Pure agar cultures were obtained by single-ascospore isolations from the respective, freshly collected specimens as previously described by Jaklitsch (2009):

Parts of stromata were crushed in sterile distilled water. The resulting suspension was transferred to cornmeal agar plates (Sigma, St. Louis, Missouri) supplemented with 2 % (w/v) D(+)-glucose-monohydrate (CMD), and 1 % (v/v) of an aqueous solution of 0.2 % (w/v) streptomycin sulfate (Sigma) and 0.2 % (w/v) neomycin sulfate (Sigma). Plates were incubated overnight at 25 °C. In order to exclude possible contamination by spores of other fungal species, few germinated ascospores from within an ascus were transferred to fresh plates of CMD using a thin platinum wire. The plates were sealed with Parafilm (Pechiney, Chicago, Illinois) and incubated at 25 °C. As all species listed in Table 2 could unambiguously be identified by their morphological and growth characteristics (Jaklitsch 2009, 2011), no molecular phylogenetic analyses needed to be performed.

Detailed descriptions of chemicals, extraction and work-up procedures for specimens and agar plate cultures, cultivation methods, as well as comprehensive protocols for HPLC/QTOF-ESI-HRMS were given by Röhrich et al. (2012, 2013a). For routine screening, a high-resolution micrOTOF Q-II mass spectrometer with orthogonal ESI source (Bruker Daltonic, Bremen, Germany), coupled to an UltiMate 3000 HPLC (Dionex, Idstein, Germany), was used. Samples, which have been screened negative with the above HPLC/MS system, were re-examined using a maXis 3G QTOF mass spectrometer with orthogonal ESI source (Bruker Daltonic, Bremen, Germany), coupled to an UltiMate 3000 UHPLC (Dionex, Idstein, Germany) as previously described (Röhrich et al. 2012, 2013a).

## Results and discussion

### General considerations

All strains investigated in this study represent phylogenetically well-defined species (Tables 2 and 3). This is in contrast to most of the reports published until the end of the 1990s, when peptaibiotic production by the genus *Trichoderma/Hypocrea* was – according to Rifai’s classification (1969) – mostly attributed to one of the four common species *T. viride*, *T. koningii, T. harzianum, T. longibrachiatum*, and sometimes to *T. pseudokoningii* and *T. aureoviride*. Careful inspection of the literature published prior to the turn of the millennium revealed that only three of the *Trichoderma* strains, reported as sources of ‘classical’ peptaibiotics have correctly been identified and appropriately been deposited, viz. the paracelsin-producing *T. reesei* QM 9414 (Brückner and Graf 1983; Brückner et al. 1984), the trichosporin/trichopolyn producer *T. polysporum* TMI 60146 (Iida et al. 1990, 1993, 1999), and the paracelsin E-producing *T. saturnisporum* CBS 330.70 (Ritieni et al. 1995). Furthermore, none of the numerous peptaibiotic-producing strains, reported to belong to those six *Trichoderma* species mentioned above, has subsequently been verified by phylogenetic analyses. Statements on the identity of the producers must therefore be regarded with great caution, unless it is being described how isolates were identified (Degenkolb et al. 2008). Unfortunately, most of the peptaibiotic-producing *Trichoderma/Hypocrea* strains investigated prior to 2000 have never been appropriately deposited either *i*) in a publicly accessible culture collection or *ii*) in an International Depositary Authority (IDA) under the conditions of the Budapest Treaty; thus, they are not available to independent academic research. As misidentifications persist to be a continuous problem, not only in the older literature (Neuhof et al. 2007), the authors prefer to introduce new names for the peptaibiotics sequenced in this study. Those new names refer to the epithets of the producing species.

### Screening of *Hypocrea thelephoricola*

**Fig. 1:**
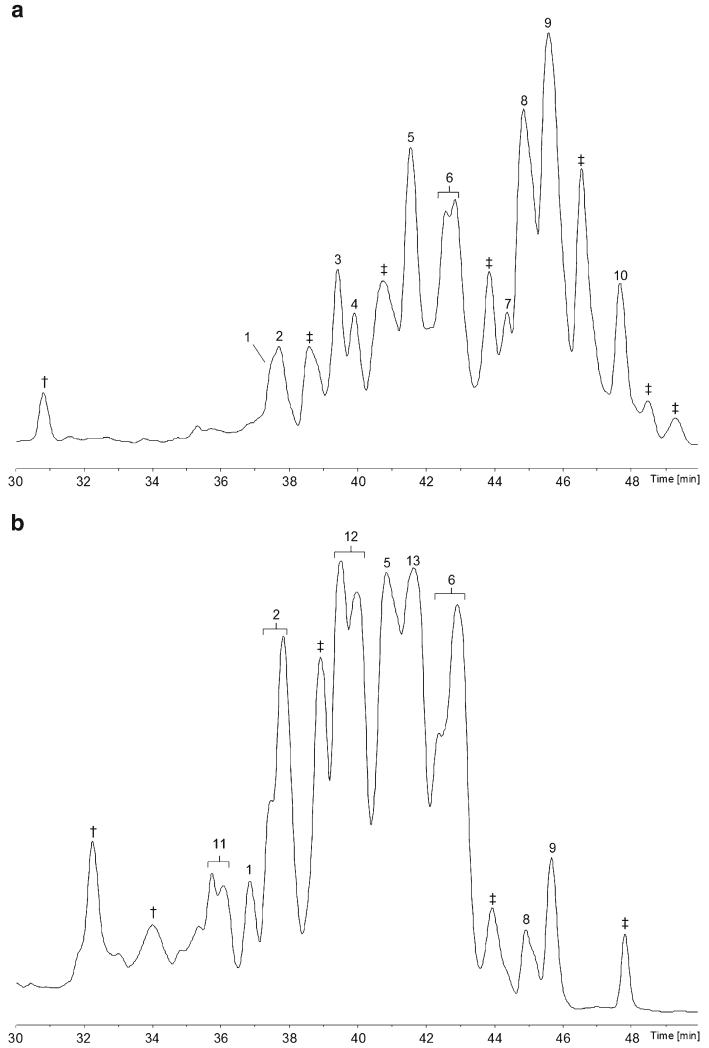
Base-peak chromatograms (BPCs) analysed with the micrOTOF-Q II **a** specimen of *H. thelephoricola*; **b** plate culture of *H. thelephoricola* on PDA. †, non-peptaibiotic metabolite(s); ‡, co-eluting peptaibiotics, not sequenced. The *y*-axis of all BPC chromatograms in this publication refers to relative ion intensities

Ten peptaibols from the specimen of *H. thelephoricola* were sequenced (Fig. 1a). Six of them, compounds **1**–**6**, are 11-residue sequences displaying the classical building scheme of subfamily 4 (SF4) peptaibols (Chugh and Wallace 2001; Degenkolb et al. 2012; Röhrich et al. 2013b). Compound **1** is new, whereas compounds **2**–**6** are likely to represent 11-residue peptaibols, which have been described before (Tables 4 and 5, Table S1a and S1b). Compounds **7**–**10** are new 18-residue peptaibols, named **thelephoricolins 1**–**4** sharing some structural similarity (*N*-terminal dipeptide, [Gln]^6^/[Aib]^7^, *C*-terminal heptapeptide) with trichotoxins A-50H and A-50-J^5^ (Brückner and Przybylski 1984). The plate culture produced predominantly 11-residue SF4-peptaibols (compounds **1**, **2**, **5**, **6**, **11**–**13**), but only two 18-residue peptaibols, **thelephoricolins 2** and **3** (Fig. 1b).

### Screening of *Hypocrea gelatinosa*

**Fig. 2:**
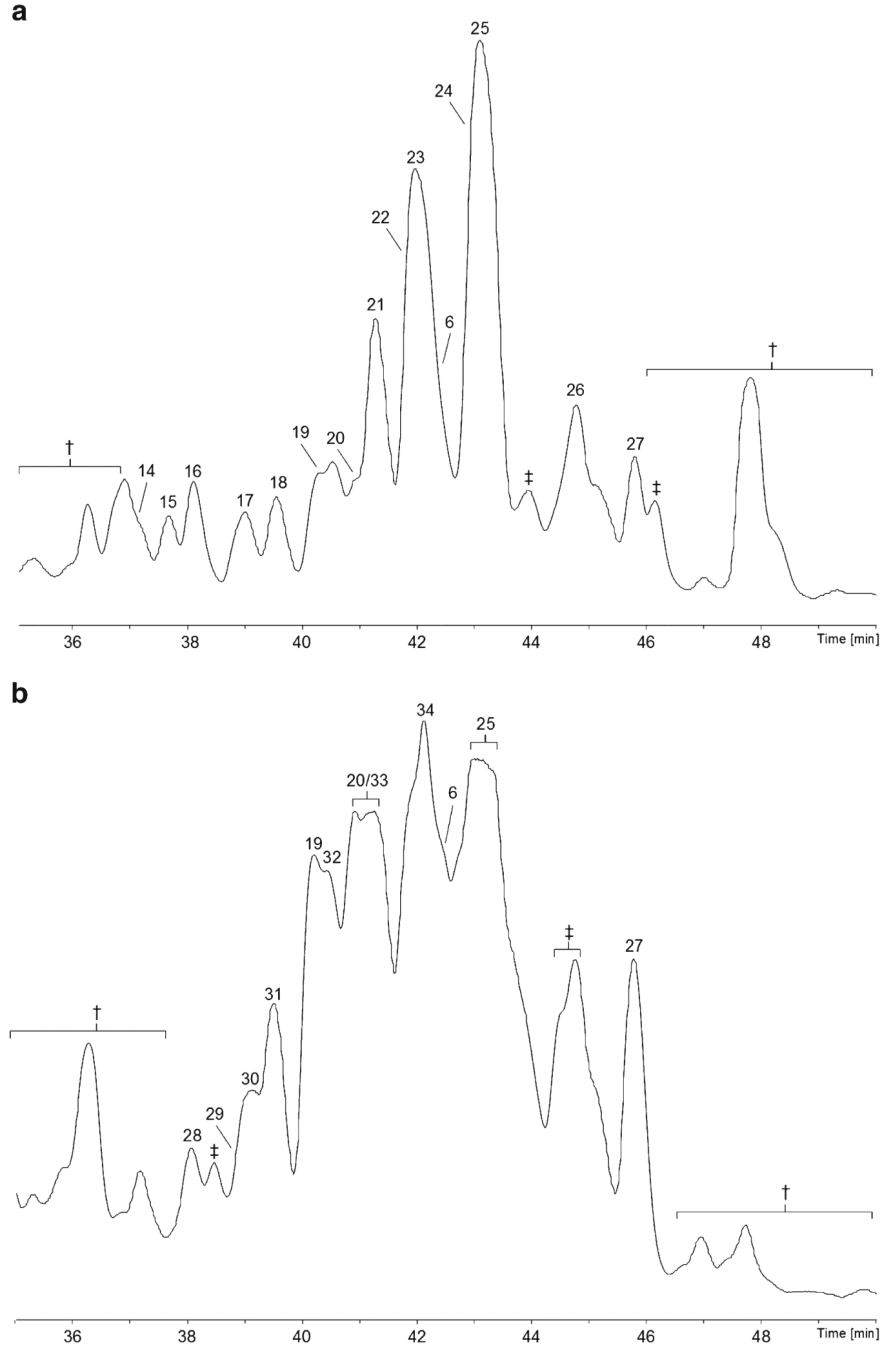
Base-peak chromatograms (BPCs) analysed with the micrOTOF-Q II **a** specimen of *H. gelatinosa*; b plate culture of *H. gelatinosa* on PDA. †, non-peptaibiotic metabolites, not sequenced; ‡, co-eluting peptaibiotics, not sequenced

A single strain (ICMP 5417) of this species has previously been screened positive Aib and Iva by a GC/MS-based approach (Brückner et al. 1991). From the specimen of *H. gelatinosa*, 14 compounds **14**–**27**, six 18-residue and eight 19-residue peptaibols, were sequenced. All of them but compounds **14** and **18** are new (Tables 6 and 7, Table S2a and S2b; Fig. 2a). The 18-residue sequences, compounds **19**–**21**, **23**, **25**, and **27**, named **gelatinosins B 1–6**, resemble hypomurocins^6^ or neoatroviridins^7^. Two of the 19-residue sequences, compounds **14** and **18**, are identical with the recently described hypopulvins from *H. pulvinata* (Röhrich et al. 2012). The new compounds **15**–**17**, **22**, and **24**, named **gelatinosins A 1–5**, exhibit a partially new building scheme – the residue in position 5 of the peptide chain was assigned as Phe, based upon HR-MS/MS data. In contrast to this, the new 19-residue compound **26** displays a different building scheme, resembling trichostrigocinsA/B (Degenkolb et al. 2006a). The plate culture of *H. gelatinosa* was shown to produce three minor 11-residue SF4-peptaibols, compounds **6**, **29**, and **33**, and nine **gelatinosins B** (compounds, **19**, **20**, **25**, **27**, **28**, **30**–**32,** and **34**), 18-residue peptaibols of the hypomurocin/neoatroviridin-type. However, 19-residue peptaibols have not been detected (Tables 6 and 7, Table S2a and S2b; Fig. 2b).

Compound **6** is likely to represent the second one of the partial sequences reported by Krause et al. (2006a) for *H. gelatinosa* CBS 724.87. In contrast, the first one, for which an unknown *N*-terminal residue *m/z* 157 was claimed (Krause et al. 2006a), could not be detected in this screening.

### Screening of *Hypocrea voglmayrii*

**Fig. 3:**
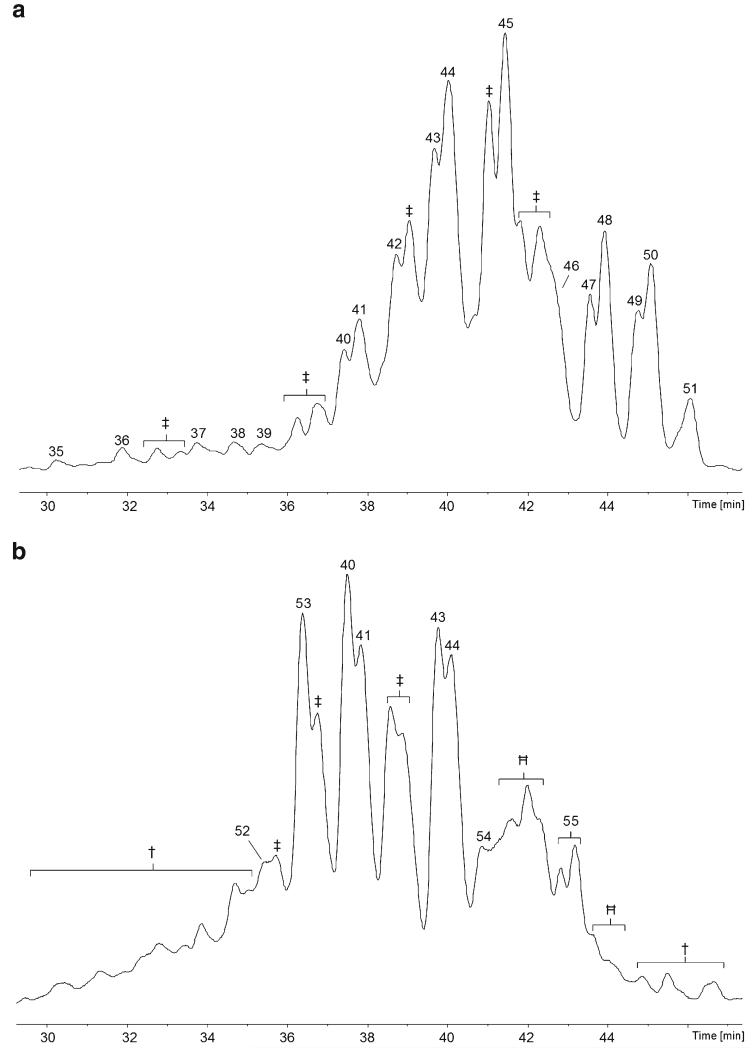
Base-peak chromatograms (BPCs) analysed with the micrOTOF-Q II **a** specimen of *H. voglmayrii*; **b** plate culture of *H. voglmayrii* on PDA. †, non-peptaibiotic metabolite(s); ‡, co-eluting peptaibiotics, not sequenced; Ħ, minor peptabiotics containing *O*-prenylated tyrosinol (Tyr(C_5_H_8_)ol), the *C*-terminus of which could not be sequenced

**Fig. 4:**
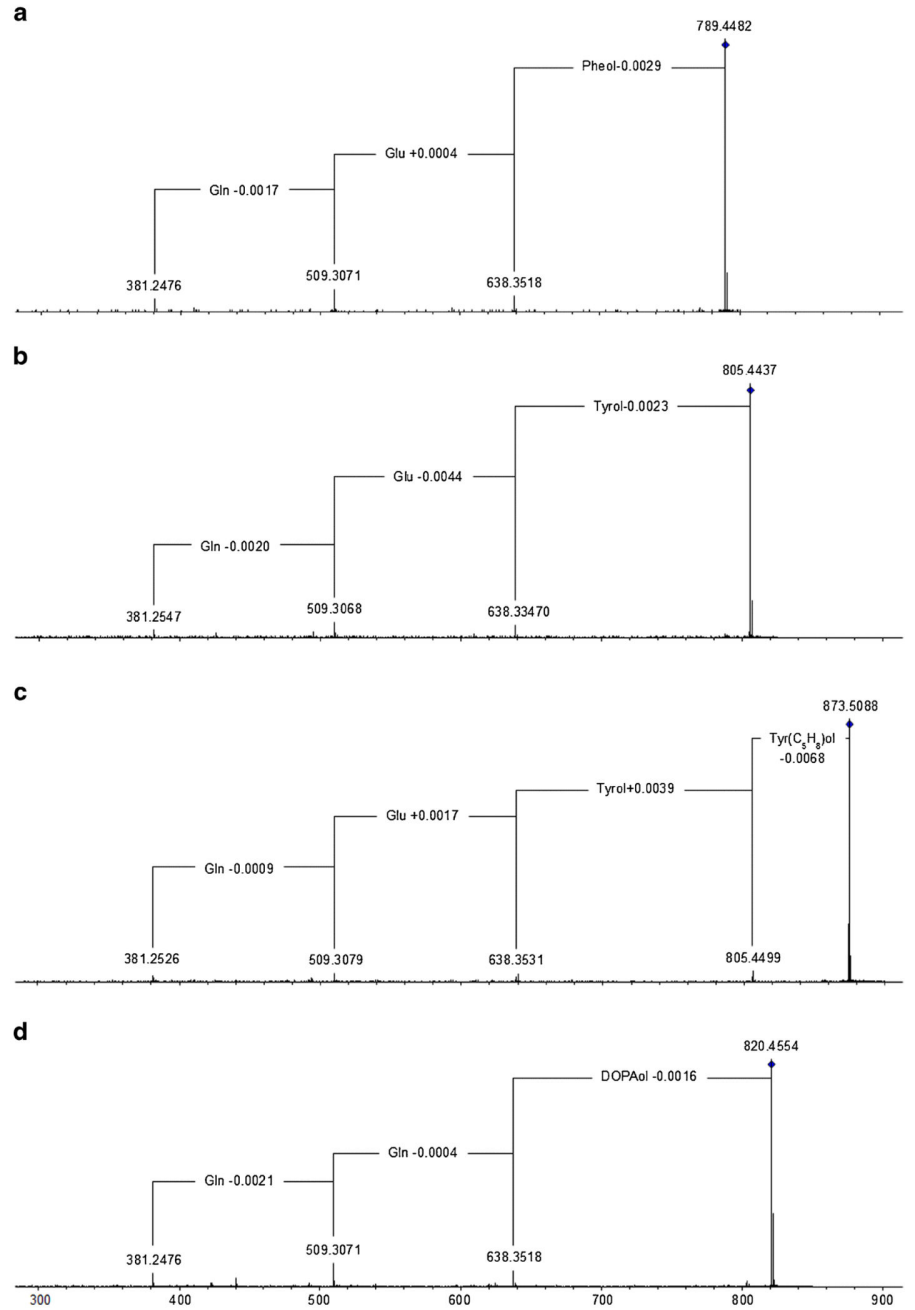
HR-MS/MS sequencing of diagnostic, *C*-terminal *y*-ions, displaying novel and recurrent residues of *β*-amino alcohols **a** phenylalaninol (Pheol); **b** tyrosinol (Tyrol); **c** *O*-prenylated tyrosinol (Tyr(C_5_H_8_)ol); **d** dihydroxyphenylalaninol (DOPAol)

The most notable species screened is by far *H. voglmayrii* (Fig. 3), the specimen of which produced two 18-residue deletion sequences, compounds **35** and **36**, which lack the *C*-terminal amino alcohol, as well as 15 19-residue peptaibols, compounds **37**–**51** (Tables 8 and 9, Table S3a and S3b). As all of them are new, the names **voglmayrins 1**–**17** are introduced. They partly resemble the building schemes of trichokonin V (Huang et al. 1995) and of trichorzianins B (Rebuffat et al. 1989). Six of the major compounds (**40**–**45**) carry a *C*-terminal phenylalaninol (Pheol) residue, whereas three minor compounds (**37**–**39**) terminate in tyrosinol (Tyrol) – a residue that has not been described for peptaibiotics until only recently (Röhrich et al. 2013a). Another six major compounds (**46**–**51**) display an additional fragment ion 68.0628 ± 2.3 mDa at their *C*-terminus (Fig. 4). Thus, the *p*-OH group of their Tyrol residue is hypothesised to be substituted by a prenyl or isoprenyl residue (C_5_H_8_, for details see paragraph below). In contrast to this, major 19-residue peptaibols produced by the plate culture, compounds **40**, **41**, **43**, **44**, and two additional compounds, **52** and **53**, **voglmayrins-18** and -**19**, terminate in Pheol. HR-MS data clearly confirm the presence of additional minor components carrying a *C*-terminal Tyrol or prenylated Tyrol residue, respectively. Unfortunately, the intensities were too low for MS/MS sequencing of the respective *y_6_* ions. Two 11-residue lipopeptaibols, compound **54** and **55**, resembling lipostrigocin B-04/B-05 (Degenkolb et al. 2006a) and trichogin A IV (Auvin-Guette et al. 1992), have also been sequenced.

### Screening of *Hypocrea minutispora*

**Fig. 5:**
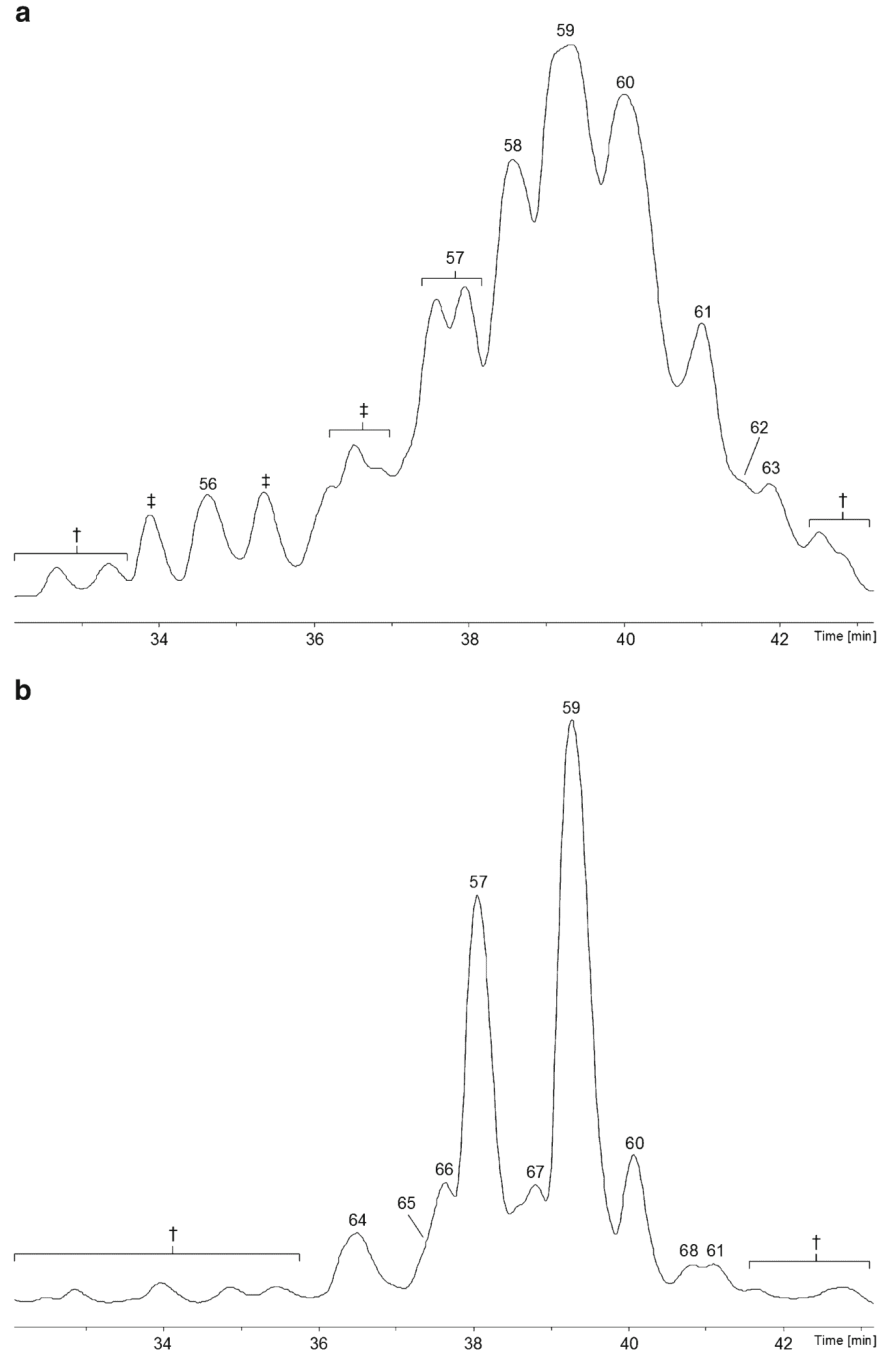
Base-peak chromatograms (BPCs) analysed with the micrOTOF-Q II **a** specimen of *H. minutispora*; b plate culture of *H. minutispora* on PDA. †, non-peptaibiotic metabolite(s); ‡, co-eluting peptaibiotics, not sequenced

The specimen of *H. minutispora* has been shown to produce a mixture of eight new 19-residue peptaibols, compounds **56**–**63**, named **minutisporins 1**–**8** (Tables 10 and 11, Table S4a and S4b; Fig. 5a), resembling the recently described hypophellins (Röhrich et al. 2013a). Analysis of the plate culture (Fig. 5b) revealed that compounds **59**–**61** were recurrently isolated along with another five new 19-residue sequences, **minutisporins 9**–**13** (compounds **64**–**68**).

### Screening of *Hypocrea citrina*

**Fig. 6:**
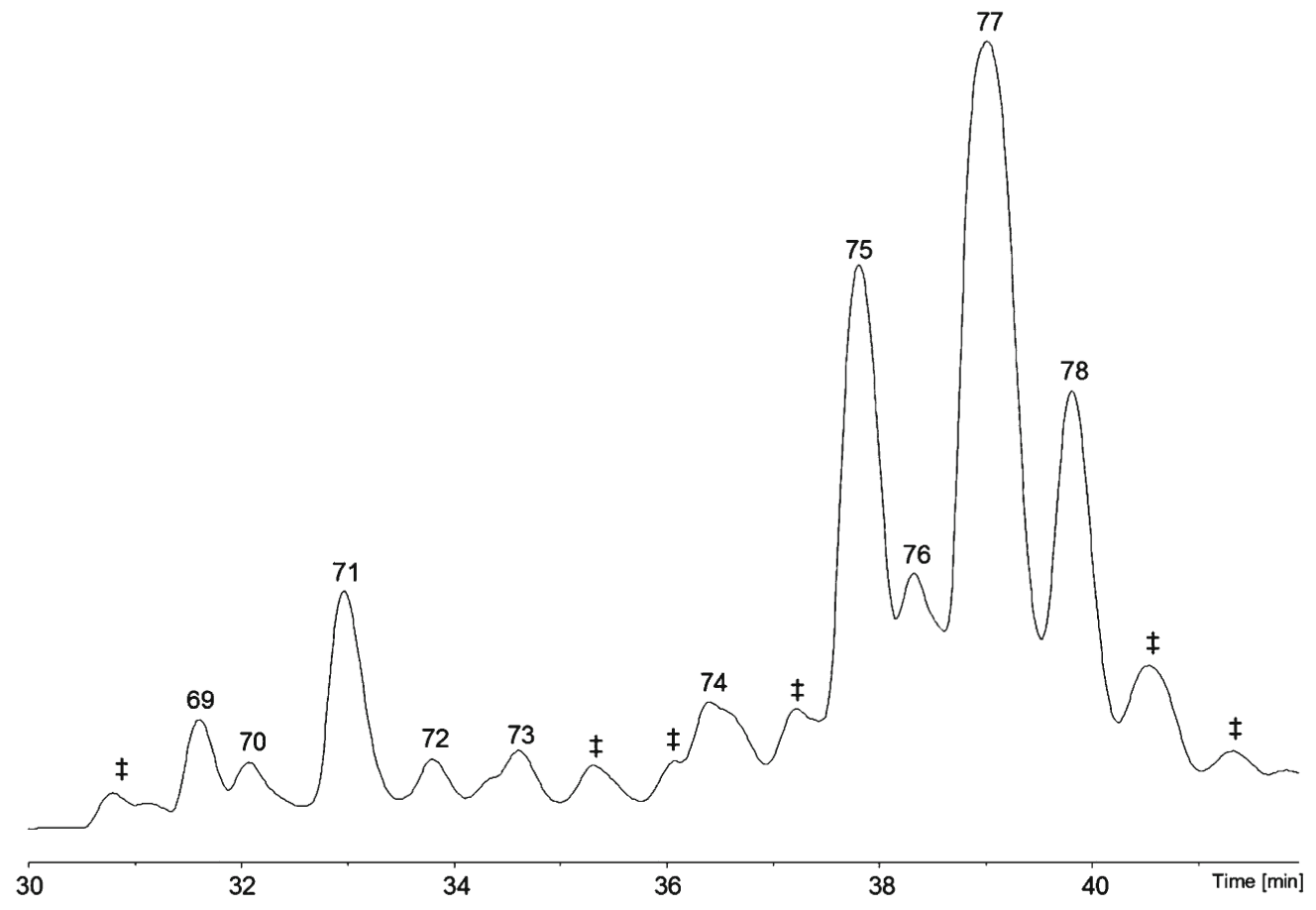
Base-peak chromatograms (BPCs) of the specimen of *H. citrina* analysed with the micrOTOF-Q II ‡, co-eluting peptaibiotics, not sequenced

The specimen of *H. citrina* was shown to be a prolific producer of 19-residue peptaibols, compounds **69**–**78**, of which seven are new, viz. compounds **69**, **70**, **72**–**74**, **76**, and **78**. The names **hypocitrins 1**–**7** were selected in order to avoid possible confusion with the mycotoxin citrinin and its derivatives. The remaining three were identified as hypophellin-15, –18, and –20, respectively (Röhrich et al. 2013a). Notably, compound **69**, **hypocitrin-1**, exhibits a *C*-terminal substituent, which is novel to peptaibiotics, dihydroxyphenylalaninol (Table 12 and Table S5; Fig. 6). Compound **70**, **hypocitrin-2**, a homologue of hypophellin-15 (compound **73**), also terminates in Tyrol (Fig. 4). Due to exceptionally high background noise of unknown origin, the methanolic extract of the well-grown *H. citrina* plate culture could not be interpreted appropriately.

### Screening of *Hypocrea sulphurea*

All three specimens of *H. sulphurea* were negatively screened for peptaibiotics. From two of them, plate cultures could be obtained; however, those were also screened negatively (data not shown).

### Screening of *Hypocrea parmastoi*

Neither specimen, nor plate culture of *H. parmastoi* displayed the presence of peptaibiotics (data not shown).

Screening of specimens collected in the natural habitat(s) corroborated the distinguished importance of the genus *Trichoderma*/*Hypocrea* as the currently richest source of peptaibiotics. Five of the nine specimens were screened positively, and the results of this screening confirmed by the sequences obtained from screening of the plate cultures. Notably, 56 of the 78 peptaibiotics (72 %) detected represent new sequences.

Screening of *H. voglmayrii* and *H. citrina* revealed five peptaibols (compounds **37**–**39**, **70**, and **73**) carrying a *C*-terminal Tyrol, a residue quite recently described for *H. phellinicola* (Röhrich et al. 2013a), which is considered comparatively rare. The additional substituent of the *C*-terminal Tyrol of voglmayrins 12–17 (compounds **46**–**51**), which has tentatively been assigned as a prenyl or isoprenyl (C_5_H_8_) residue, is hypothesised to be located at the *p*-hydroxy group. A regiospecific *O*-prenylation at the 4-position of the aromatic ring has recently been demonstrated for SirD (Zou et al. 2011), a tyrosine *O*-prenyltranferase (Kremer and Li 2010) catalysing the first pathway-specific step in the biosynthesis of the phytotoxin sirodesmin PL. The latter is produced by *Leptosphaeria maculans* (anamorph: *Phoma lingam*), the causal agent of blackleg of canola (*Brassica napus*). Recently, *O*-prenyltyrosine diketopiperazines have been described from *Fusarium* sp. and *Penicillium crustosum* (Guimarães et al. 2010).

Another notable structural element, dihydroxy-Pheol was found at the *C*-terminus of hypocitrin-1 (compound **69**). While the presence of either Pheol or Tyrol may be assumed to originate from the relaxed substrate specificity in the terminal adenylate domain of the respective peptaibol synthetase, the direct incorporation of dihydroxy-Phe, presumably 3,4-dihydroxy-L-Phe (DOPA), is one possible biosynthetic route. Fungal tyrosinases are known to oxidise not only Tyr and various other monophenols, e.g. in the route to melanins, but also act on tyrosyl residues within peptides and proteins, leading to the formation of inter- and intra-molecular crosslinks (Selinheimo et al. 2007). Thus, Tyrol-containing peptaibols could be further oxidised by tyrosinases, and even become attached to components of the fungal cell wall (Mattinen et al. 2008).

Considering the sequences of all species screened, including those of *H. pulvinata* and *H. phellinicola*, a general building scheme for those SF1-peptaibiotics can be given (Table 13):

As can be seen from above, all structural features (Röhrich et al. 2012) required for ion channel formation (Grigoriev et al. 2003), are present in the 17-, 18-, 19-, and 20-residue peptaibiotics sequenced. Multiple bioactivities of pore-forming 20-residue SF1-peptaibiotics (Röhrich et al. 2013a) and of 11-residue SF4-peptaibiotics (Bobone et al. 2013; Röhrich et al. 2013b) have recently been compiled.

The results of our screening programme further extend the list of peptaibiotic-producing species of *Trichoderma/Hypocrea* compiled in Table 14. Most notably, the sequences of peptaibiotics produced by the freshly collected specimens are either identical to those found in the plate cultures, or represent – at least – closely related homologues and positional isomers of the latter. Thus, our LC-MS/MS screening approach confirmed that all peptaibiotic-producing specimens and plate cultures obtained thereof represent one and the same species. Consequently, the same type (= subfamily) of peptaibiotics is produced both in the natural habitat and under artificial (= laboratory) conditions – a fact, which is important for the application of *Trichoderma* formulations in biocontrol and integrated pest management schemes. A*Trichoderma*/*Hypocrea* species capable of producing peptaibiotics under the conditions of its natural habitat may defend its ecological niche more effectively compared to a non-producing species, as will be outlined below. At present, ca. 15 % of the phylogenetically verified *Trichoderma*/*Hypocrea* species have been positively screened for peptaibiotics; however, it appears that the inventory of peptaibiotics of the remaining 85 % is still waiting to be scrutinised by state-of-the-art bioanalytical – particularly mass spectrometric – methods. Of approximately 130 *Trichoderma*/*Hypocrea* species pre-screened by LC/HRMS (Nielsen et al. 2011), ca. 60 were found to produce peptaibiotics^8^. Thus, the production of peptaibiotics in the natural habitat seems to be independent of the habitat preference, i.e. mycoparasitism vs. saprotrophy (Chaverri and Samuels 2013), but neither predictable per se nor universal.

Given that peptaibiotics are readily biosynthesised in the natural habitat of the producers, they could significantly contribute to the complex interactions of phytoprotective *Trichoderma* species, which are used in commercial or semi-commercial biocontrol agents (BCAs) against plant pathogenic fungi (Harman et al. 2004; Viterbo et al. 2007; Vinale et al. 2008a, b). Examples of successful biocontrol approaches using *Trichoderma* strains include ‘*Tricovab*’, a Brazilian formulation recently approved (Anonymous 2012) for integrated management of *Crinipellis* (syn. *Moniliophthora*) *perniciosa*, the causal agent of Witches’ broom of cacao (Pomella et al. 2007; Loguercio et al. 2009; Medeiros et al. 2010). Notably, ‘*Tricovab*’ contains a peptaibiotic-producing strain (Degenkolb et al. 2006a) of the hyperparasitic endophyte *Trichoderma stromaticum*. Moreover, the in vivo-detection of peptaibiotics corroborates the recently demonstrated pro-apoptotic in vitro-activities of the 19-residue peptaibols trichokonin VI^9^ (Huang et al. 1995) from *Trichoderma pseudokoningii* SMF2 towards plant fungal pathogens such as *Fusarium oxysporum* (Shi et al. 2012).

The value of peptaibiotics for chemotaxonomy of *Trichoderma*/*Hypocrea* has scarcely been scrutinised in the past (Neuhof et al. 2007; Degenkolb et al. 2008). To exhaustively answer this question, a larger number of strains, belonging to recently described species, are required to be included in an LC-MS/MS-based study aimed at analysing the peptaibiome of strains and species within different clades of *Trichoderma*/*Hypocrea*. However, statements on peptaibiotic production by a particular *Trichoderma/Hypocrea* species must always be treated with great caution as they are highly habitat-, isolate-, and/or cultivation-dependent. Furthermore, ‘peptaibol subfamilies’ were introduced at a time when the total number of peptaibiotics described did not exceed 200 (Chugh and Wallace 2001) – less than a sixth of the currently known sequences. Notably, the additional 1,000–1,100 individual peptaibiotics published since then exhibit both new building schemes and constituents. This issue becomes even more complex as ‘peptaibol subfamilies’ were published when phylogenetic methods have not yet been recognised as an indispensable tool in fungal taxonomy. Thus, a considerable number of peptaibiotics, the sequences of which have been elucidated correctly, cannot be linked to an unambiguously identified producer that is deposited in a publicly accessible culture collection. These facts illustrate the urgent need to reconsider the classification into the nine subfamilies – a task that has to be completed before the aforementioned study can be performed.

Currently, any approach for a peptaibiotics-based chemotaxonomy of *Trichoderma*/*Hypocrea* must be regarded as extremely complicated – even within a defined clade –, because *i*) peptaibiotics only represent one single class of secondary metabolites produced by *Trichoderma*/*Hypocrea*, *ii*) most of the producers reported in literature have never been deposited appropriately, and *iii*) the persistently high degree of misidentification makes any comparison between members of different clades problematic and challenging. This is illustrated by the following examples (references are compiled in Table 14):

- The 20-residue alamethicins (ALMs) have hitherto been found in four species belonging to the Brevicompactum clade of *Trichoderma*; however, it is not yet possible to estimate if the Pro^2^ residue of the ALMs could be regarded as a structurally highly conserved position, comparable to the Pro^14^ residue. Chemotaxonomy of the Brevicompactum clade encompassed the comparison of hydrophobins, peptaibiotics, and low-molecular weight secondary metabolites, including simple trichothecene-type mycotoxins.
- The 18-residue trichotoxins (TXT) A-50 and A-40, for example, have been obtained from *Trichoderma asperellum* NRRL 5242, whereas *Trichoderma asperellum* Y 19-07 did not produce TXTs but 9- and 10-residue peptaibols instead (and vice versa).
- *Trichoderma citrinoviride* strains S 25 and IMI 91968 are rich sources of 20-residue peptaibols of the paracelsin/saturnisporin/trichocellin/suzukacillin/trichoaureocin-type. These are the only two strains of *T. citrinoviride* that have been investigated for peptaibiotics. *Hypocrea schweinitzii* ICMP 5421, which has also been verified phylogenetically (Réblová and Seifert 2004), had only been screened positive for Aib by GC/MS; but – to the best of the authors’ knowledge – specimens of that species have never been investigated for its inventory of peptaibiotics. Parcelsins, which have been isolated from *T. reesei* QM 9414, are also produced by a member of the Longibrachiatum clade. However, the producer of saturnisporin (*T. saturnisporum* MNHN 903578: Rebuffat et al. 1993) has never been made publicly available, nor has its identity been verified phylogenetically. The producers of both trichocellins and suzukacillins A (Krause et al. 2006b) have not been deposited in a publicly available culture collection; thus, their identification as *T*. ‘*viride*’ is highly questionable.
- *T. flavofuscum* CBS 248.59 is the only species of *Trichoderma*/*Hypocrea*, which produces 13-residue sequences – notably trichofumins C and D are the only two peptaibols of that chain length reported to date. They display the rare Gln-Gln motif in positions 5 and 6. Looking at the sequences, their biosynthesis seems to be distantly related to that one of trichofumins A and B (and positional isomers thereof). The latter are 11-residue SF4-peptaibols and widespread amongst *Trichoderma*/*Hypocrea* species.
- *T. virens* strain Tv29-8 produces common 11- and 14-residue peptaibols, and it is the only phylogenetically verified source of 18-residue peptaibols of the trichorzin-type.

However, the results of our LC-MS/MS screening are also of interest for analysis of environmental samples as well as extraterrestrial materials such as carbonaceous meteorites as their contamination by propagules of soil- or airborne peptaibiotic-producing fungi has to be taken into account (Brückner et al. 2009; Elsila et al. 2011).

To sum up, production of peptaibiotics may generally be regarded as a sophisticated ecological adaptation for the producing fungus providing it with an obvious advantage over non-producing fungal and other competitors. This group of ‘*chemical weapons*’ in their ‘*armoury*’ may effectively assist a remarkable number of strains currently identified as belonging to ca. 30 *Trichoderma/Hypocrea* species in colonising and defending their ecological niches.

## Supplementary Material

Supplementary Tables 1-9

## Figures and Tables

**Table 1:** Recently described, non-peptaibiotic secondary metabolites from *Trichoderma/Hypocrea* species not yet listed in AntiBase 2013

Producing species and strains	Name of new metabolite(s)	Chemical subclass of metabolites	References
*T. atroviride* G20-12	4′-(4,5-dimethyl-1,3-dioxolan-2-yl)methylphenol (3′-hydroxybutan-2′-yl)5-oxopyrrolidine-2-carboxylate Atroviridetide		Lu et al. 2012
*T atroviride* UB-LMA^a^	one bicyclic, three tetracyclic diterpenes	Di- and tetraterpenes	Adelin et al. 2014
*T gamsii* SQP 79–1	Trichalasin C, D	Cytochalasans	Ding et al. 2012
		Spiro-cytochalasan	Ding et al. 2014
*T.* sp. FKI-6626	Cytosporone S		Ishii et al. 2013
*T. erinaceum* AF007	Trichodermaerin	Diterpenoid lactone	Xie et al. 2013

a The scientific name of the producer has been misspelled as *Trichoderma atroviridae* in Adelin et al. (2014)

**Table 2:** Habitat and geographic distribution of *Hypocrea* species included in this study

Species	Clade	Habitat	Geographic distribution
*Hypocrea thelephoricola (Trichoderma thelephoricola)*	Chlorospora	On and around basidiomata of *Steccherinum ochraceum*, on wood and bark	North America (USA), Europe (Austria)
*Hypocrea minutispora (Trichoderma minutisporum)*	Pachybasium (core group)	Most common hyaline-spored species in temperate zones	Europe (Austria, Czech Republic, Denmark, Estonia, France, Germany, Spain, Sweden, United Kingdom) and North America (USA)
*Hypocrea sulphurea (Trichoderma* sp.)	Hypocreanum	On basidiomes of *Exidia* spp.	Europe (Eastern Austria, Ukraine), North America (USA), Japan
*Hypocrea citrina (Trichoderma lacteum)*	Hypocreanum	Spreading from stumps or tree bases on soil and debris such as small twigs, bark, leaves, dead plants; incorporating also living plants; more rarely on bark of logs on the ground. Most typically in mixed coniferous forest	widespread and locally common, mostly found from the end of August to the beginning of October. Europe (Austria, Belgium, Czech Republic, Netherlands, Sweden, United Kingdom) and North America (USA)
*Hypocrea voglmayrii (Trichoderma voglmayrii)*	Lone lineage	On dead, mostly corticated branches and small trunks of *Alnus alnobetula (= A. viridis) and A. incana* standing or lying on the ground	Austria (at elevations of 1,000–1,400 m in the upper montane vegetation zone of the Central Alps)
*Hypocrea gelatinosa (Trichoderma gelatinosum)*	Lone lineage	On medium- to well-decayed wood, also on bark and overgrowing various fungi	Europe (Austria, France, Germany, Netherlands, Slovenia, Ukraine, United Kingdom)
*Hypocrea parmastoi (Trichoderma* sp. [sect. Hypocreanum])	Lone lineage	On medium- to well-decayed wood and bark of deciduous trees	Europe (Austria, Estonia, Finland, France, Germany); uncommon

Data were compiled from Chaverri and Samuels (2003), Overton et al. (2006a, b), and Jaklitsch (2009, 2011)

**Table 3:** Habitat and geographic origin of *Hypocrea* isolates included in this study

Isolate	Substrate	Collecting information	Culture
*H. thelephoricola*	*Steccherinum ochraceum/Carpinus betulus*	Austria, Niederosterreich, Wien-Umgebung, Mauerbach, MTB 7763/1, 13 June 2011,W. Jaklitsch	CBS 133226
*H. gelatinosa*	*Carpinus betulus*		CBS 133223
*H. minutispora*	*Carpinus betulus*		CBS 133224
*H. sulphurea* 1	*Exidia glandulosalCarpinus betulus*		not deposited^a^
*H. sulphurea* 2^b^	*Exidia glandulosal Carpinus betulus*		CBS 133227
*H. sulphurea* 3	*Exidia* sp.	Austria, Vienna, Lainzer Tiergarten, near Nikolaitor, 25 September 2011, H. Voglmayr	not deposited
*H. parmastoi*	*Fagus sylvatica*	Austria, Niederosterreich, Wien-Umgebung, Mauerbach, MTB 7763/1,30 October 2011, W. Jaklitsch (Hypo 656)	CBS 133242
*H. voglmayrii*	*Alnus alnobetula*	Austria, Styria, Schladming, Untertal, at Riesachfalle, 12 June 2011, H. Voglmayr	CBS 133225
*H. citrina*	*Pinus sylvestris* litter, ground	Austria, Carinthia, Obermieger, Sabuatach, MTB 9452/2, 23 September 2011, W. Jaklitsch (Hypo 654)	CBS 133244

a Stroma immature, isolation of single germinable ascospores impossible

b The specimens of *H. sulphurea* 1 and 2 were collected from two different trees found in the same area

**Table 4:** Sequences of 11- and 18-residue peptaibiotics detected in the specimen of *Hypocrea thelephoricola*

No.	t_R_ [min]	[*M*+ H]^+^		Residue^a^																	
				1	2	3	4	5	6	7	8	9	10	11	12	13	14	15	16	17	18
1	37.6–37.9	1161.7527	Ac	Aib	Gln	Vxx	Lxx	Aib	Pro	Vxx	Lxx	Aib	Pro	Lxxol							
2	37.6–37.9	1161.7527	Ac	Aib	Gln	Vxx	Vxx	Aib	Pro	Lxx	Lxx	Aib	Pro	Lxxol							
3	39.3–39.5	1175.7712	Ac	Aib	Gln	Vxx	Lxx	Aib	Pro	Lxx	Lxx	Aib	Pro	Lxxol							
4	39.7 –40.0	1175.7712	Ac	Aib	Gln	Lxx	Lxx	Aib	Pro	Vxx	Lxx	Aib	Pro	Lxxol							
5	41.5–1.7	1189.7836	Ac	Aib	Gln	Lxx	Lxx	Aib	Pro	Lxx	Lxx	Aib	Pro	Lxxol							
6	42.9–3.0	1203.7981	Ac	Vxx	Gln	Lxx	Lxx	Aib	Pro	Lxx	Lxx	Aib	Pro	Lxxol							
7	44.2–4.5	1732.0673	Ac	Aib	Ala	Aib	Ala	Vxx	Gln	Aib	Vxx	Aib	Gly	Lxx	Aib	Pro	Lxx	Aib	Vxx	Gln	Vxxol
8	44.8–5.0	1746.0866	Ac	Aib	Ala	Aib	Ala	Vxx	Gln	Aib	Lxx	Aib	Gly	Lxx	Aib	Pro	Lxx	Aib	Vxx	Gln	Vxxol
9	45.2–6.0	1760.1035	Ac	Aib	Ala	Vxx	Ala	Vxx	Gln	Aib	Lxx	Aib	Gly	Lxx	Aib	Pro	Lxx	Aib	Vxx	Gln	Vxxol
10	47.5–7.8	1774.1161	Ac	Aib	Ala	Vxx	Ala	Vxx	Gln	Aib	Lxx	Aib	Gly	Lxx	Aib	Pro	Lxx	Aib	Vxx	Gln	Lxxol

No.	Compound identical or positionally isomeric with	Ref.																			
1	New																				
2	Trichorovins: Ilia, IVa	Wada et al. 1995																			
	Hypomurocin A-l	Becker et al. 1997																			
	Trichobrachins III: 5, 9b	Krause et al. 2007																			
	Tv-29-ll-III g	Mukherjee et al. 2011																			
	Hypojecorin A: 8	Degenkolb et al. 2012																			
3	Trichobrachins III: 10a, 12a, 15b	Krause et al. 2007																			
	Trichorovins: VTII, IXa	Wada et al. 1995																			
	Hypomurocin A-3	Becker et al. 1997																			
	Tv-29-11-IV g	Mukherjee et al. 2011																			
4	Tv-29-11-IV e	Mukherjee et al. 2011																			
5	Trichobrachins III: 16a, 17, 18	Krause et al. 2007																			
	Trichorovins: XIII, XIV	Wada et al. 1995																			
	Tv-29-11-V b	Mukherjee et al. 2011																			
	Hypomurocins: A-5, A-5a	Becker et al. 1997																			
	Trichorozin IV	Iida et al. 1995																			
	Trichobrachins: C-I, C-II	Ruiz et al. 2007																			
	Trilongin A0	Mikkola et al. 2012																			
6	Trichofumin B	Berg et al. 2003																			
	Tv-29-ll-VI	Mukherjee et al. 2011																			
7	Tlielephoricolin-1																				
8	Tlielephoricolin-2																				
9	Tlielephoricolin-3																				
10	Tlielephoricolin-4																				

a Variable residues are underlined in the table header. Minor sequence variants are underlined in the sequences. This applies to all sequence tables

**Table 5:** Sequences of 11- and 18-residue peptaibiotics detected in the plate culture of *Hypocrea thelephoricola*

No.	t_R_ [min]	[*M*+H]^+^		Residue^a^																	
				1	2	3	4	5	6	7	8	9	10	11	12	13	14	15	16	17	18
11	35.6–35.8	1147.7443	Ac	Aib	Gln	Vxx	Vxx	Aib	Pro	Vxx	Lxx	Aib	Pro	Lxxol							
1	37.2–37.4	1161.7623	Ac	Aib	Gln	Vxx	Lxx	Aib	Pro	Vxx	Lxx	Aib	Pro	Lxxol							
2	37.7–37.9	1161.7652	Ac	Aib	Gln	Vxx	Vxx	Aib	Pro	Lxx	Lxx	Aib	Pro	Lxxol							
12	39.8–10.0	1175.7747	Ac	Aib	Gln	Lxx	Vxx	Aib	Pro	Lxx	Lxx	Aib	Pro	Lxxol							
5	41.5–11.7	1189.7893	Ac	Aib	Gln	Lxx	Lxx	Aib	Pro	Lxx	Lxx	Aib	Pro	Lxxol							
13	40.6–10.8	1189.7996	Ac	Vxx	Gln	Vxx	Lxx	Aib	Pro	Lxx	Lxx	Aib	Pro	Lxxol							
6	42.8–13.0	1203.8004	Ac	Vxx	Gln	Lxx	Lxx	Aib	Pro	Lxx	Lxx	Aib	Pro	Lxxol							
8	44.8–14.9	1746.0955	Ac	Aib	Ala	Aib	Ala	Vxx	Gln	Aib	Lxx	Aib	Gly	Lxx	Aib	Pro	Lxx	Aib	Vxx	Gln	Vxxol
9	45.5–15.7	1760.1104	Ac	Aib	Ala	Vxx	Ala	Vxx	Gln	Aib	Lxx	Aib	Gly	Lxx	Aib	Pro	Lxx	Aib	Vxx	Gln	Vxxol

No.	Compound identical or positionally isomeric with	Ref.																			
11	Tv-29-11-Π h	Mukherjee et al. 2011																			
1																					
2																					
12	Trichobrachin III 11a	Krause et al. 2007																			
	Tv-29-ll-IV f	Mukherjee et al. 2011																			
	Trichorovin Xa	Wada et al. 1995																			
	Hypomurocin A-4	Becker et al. 1997																			
5	cf. 2																				
13	Tv-29-ll-V d	Mukherjee et al. 2011																			
6																					
8	Thelephoricolin-2																				
9	Thelephoricolin-3																				

a Variable residues are underlined in the table header. Minor sequence variants are underlined in the sequences. This applies to all sequence tables

**Table 6:** Sequences of 11-, 18, and 19-residue peptaibiotics detected in the specimen of *Hypocrea gelatinosa*

No.	t_R_ [min]	[*M*+H]^+^		Residue^a^																		
				1	2	3	4	5	6	7	8	9	10	11	12	13	14	15	16	17	18	19
14	37.1–37.3	1866.0929	Ac	Aib	Ala	Ala	Ala	Aib	Gln	Aib	Lxx	Aib	Gly	Lxx	Aib	Pro	Vxx	Aib	Aib	Gln	Gln	Pheol
15	37.7–37.8	1895.1067	Ac	Aib	Ala	Aib	Aib	Phe	Gln	Aib	Aib	Aib	Gly	Lxx	Aib	Pro	Vxx	Aib	Aib	Glu	Gln	Lxxol
16	38.0–38.2	1908.1358	Ac	Aib	Ala	Aib	Aib	Phe	Gln	Aib	Aib	Aib	Gly	Lxx	Aib	Pro	Lxx	Aib	Aib	Gln	Gln	Lxxol
17	38.8–38.9	1909.1186	Ac	Aib	Ala	Aib	Aib	Phe	Gln	Aib	Aib	Aib	Gly	Lxx	Aib	Pro	Lxx	Aib	Aib	Glu	Gln	Lxxol
18	39.5–39.6	1880.1083	Ac	Aib	Ala	Ala	Ala	Aib	Gln	Aib	Lxx	Aib	Ala	Lxx	Aib	Pro	Vxx	Aib	Aib	Gln	Gln	Pheol
19	40.2–40.4	1762.0856	Ac	Aib	Ser	Ala	Lxx	Aib	Gln	Aib	Lxx	Aib	Gly	Vxx	Aib	Pro	Lxx	Aib	Aib	Gln	–	Lxxol
20	40.9–41.1	1762.0840	Ac	Aib	Ser	Ala	Lxx	Aib	Gln	Vxx	Lxx	Aib	Gly	Vxx	Aib	Pro	Lxx	Aib	Aib	Gln	–	Vxxol
21	41.2–41.4	1776.1023	Ac	Aib	Ser	Ala	Lxx	Vxx	Gln	Aib	Lxx	Aib	Gly	Vxx	Aib	Pro	Lxx	Aib	Aib	Gln	–	Lxxol
22	41.9	1952.1674	Ac	Aib	Ala	Aib	Aib	Phe	Gln	Aib	Aib	Aib	Ser	Lxx	Aib	Pro	Lxx	Vxx	Aib	Gln	Gln	Lxxol
23	42.1–42.3	1776.1023	Ac	Aib	Ser	Ala	Lxx	Vxx	Gln	Vxx	Lxx	Aib	Gly	Vxx	Aib	Pro	Lxx	Aib	Aib	Gln	–	Vxxol
6	42.3	1203.8117	Ac	Vxx	Gln	Lxx	Lxx	Aib	Pro	Lxx	Lxx	Aib	Pro	Lxxol								
24	42.9	1953.1515	Ac	Aib	Ala	Aib	Aib	Phe	Gln	Aib	Aib	Aib	Ser	Lxx	Aib	Pro	Lxx	Vxx	Aib	Glu	Gln	Lxxol
25	43.0–43.1	1790.1199	Ac	Aib	Ser	Ala	Lxx	Vxx	Gln	Vxx	Lxx	Aib	Gly	Vxx	Aib	Pro	Lxx	Aib	Aib	Gln	–	Lxxol
26	44.6	1919.1568	Ac	Aib	Ala	Aib	Aib	Lxx	Gln	Aib	Aib	Aib	Ser	Lxx	Aib	Pro	Vxx	Aib	Lxx	Glu	Gln	Lxxol
27	45.8	1774.1299	Ac	Aib	Ala	Ala	Lxx	Vxx	Gln	Vxx	Lxx	Aib	Gly	Vxx	Aib	Pro	Lxx	Aib	Aib	Gln	–	Lxxol

No.	Compound identical or positionally isomeric with	Ref.																				
14	Hypopulvin-9	Röhrich et al. 2012																				
15	Gelatinosin-A 1 (*C*-terminal undecapeptide cf. hypelcins B-I and -II)	Matsuura et al. 1994																				
16	Gelatinosin-A 2 (*C*-temiinal nonapeptide cf. tricholongin B-I)	Rebuffat et al. 1991																				
17	Gelatinosin-A 3 (cf. 16)																					
18	Hypopulvin-14	Röhrich et al. 2012																				
19	Gelatinosin-B 1 (cf. hypomurocin B-5: [Vxx]^8^→[Lxx]^8^)	Becker et al. 1997																				
20	Gelatinosin-B 2 (cf. hypomurocin B-3b: [Vxx]^8^→[Lxx]^8^, [Aib]^11^→ [Vxx]^11^)	Becker et al. 1997																				
21	Gelatinosin-B 3 (cf. neoatroviridin B: [Gly]^2^→ [Ser]^2^)	Oh et al. 2005																				
22	Gelatinosin-A 4 (cf. 16: [Gly]^10^→[Ser]^10^, [Aib]^15^→[Vxx]^15^)																					
23	Gelatinosin-B 4 (cf. hypomurocin B 4: [Aib]^5,7^→ [Vxx]^5,7^)	Becker et al. 1997																				
6	See *H. thelephoricola*																					
24	Gelatinosin-A 5 (cf. 17: [Gly]^10^→[Ser]^10^, [Aib]^15^→[Vxx]^15^)																					
25	Gelatinosin-B 5 (cf. neoatroviridin D: [Gly]^2^→ [Ser]^2^)	Oh et al. 2005																				
26	New (cf. trichostrigocin-A and -B: [Lxx]^16^→ [Vxx]^16^, [Gln]^17^→ [Glu]^17^)	Degenkolb et al. 2006a, b																				
27	Gelatinosin-B 6 (cf. neoatroviridin D: [Gly]^2^→[Ala]^2^)	Oh et al. 2005																				

a Variable residues are underlined in the table header. Minor sequence variants are underlined in the sequences. This applies to all sequence tables

**Table 7:** Sequences of 11- and 18-residue peptaibiotics detected in the plate culture of *Hypocma gelatinosa*

No.	t_R_ [min]	[*M*+H]^+^		Residue^a^																	
				1	2	3	4	5	6	7	8	9	10	11	12	13	14	15	16	17	18
28	38.0–38.1	1748.0789	Ac	Aib	Ser	Ala	Lxx	Aib	Gln	Aib	Lxx	Aib	Gly	Aib	Aib	Pro	Lxx	Aib	Aib	Gln	Lxxol
29	38.8–38.9	1175.7832	Ac	Aib	Gln	Lxx	Lxx	Aib	Pro	Vxx	Lxx	Aib	Pro	Lxxol							
30	39.2–39.3	1748.0789	Ac	Aib	Ser	Ala	Lxx	Aib	Gln	Aib	Lxx	Aib	Gly	Vxx	Aib	Pro	Lxx	Aib	Aib	Gln	Vxxol
31	39.4–39.7	1762.0802	Ac	Aib	Ser	Ala	Lxx	Aib	Gln	Vxx	Lxx	Aib	Gly	Aib	Aib	Pro	Lxx	Aib	Aib	Gln	Lxxol
19	40.1–40.4	1762.0814	Ac	Aib	Ser	Ala	Lxx	Aib	Gln	Aib	Lxx	Aib	Gly	Vxx	Aib	Pro	Lxx	Aib	Aib	Gln	Lxxol
32	40.5–40.7	1777.0993	Ac	Aib	Ser	Ala	Lxx	Vxx	Gln	Vxx	Lxx	Aib	Gly	Aib	Aib	Pro	Lxx	Aib	Aib	Glu	Lxxol
33	40.8–41.0	1189.8026	Ac	Aib	Gln	Lxx	Lxx	Aib	Pro	Lxx	Lxx	Aib	Pro	Lxxol							
20	40.9–41.1	1762.0797	Ac	Aib	Ser	Ala	Lxx	Aib	Gln	Vxx	Lxx	Aib	Gly	Vxx	Aib	Pro	Lxx	Aib	Aib	Gln	Vxxol
34	41.8–42.1	1776.1016	Ac	Aib	Ser	Ala	Lxx	Aib	Gln	Vxx	Lxx	Aib	Gly	Vxx	Aib	Pro	Lxx	Aib	Aib	Gln	Lxxol
6	42.7–42.9	1203.8234	Ac	Vxx	Gln	Lxx	Lxx	Aib	Pro	Lxx	Lxx	Aib	Pro	Lxxol							
25	43.M3.3	1790.1139	Ac	Aib	Ser	Ala	Lxx	Vxx	Gln	Vxx	Lxx	Aib	Gly	Vxx	Aib	Pro	Lxx	Aib	Aib	Gln	Lxxol
27	45.7–46.0	1774.1162	Ac	Aib	Ala	Ala	Lxx	Vxx	Gln	Vxx	Lxx	Aib	Gly	Vxx	Aib	Pro	Lxx	Aib	Aib	Gln	Lxxol

No.	Compound identical or positionally isomeric with	Ref.																			
28	Gelatinosin-B 7 (cf. hypomurocin B-2: [Vxx]^8^→[Lxx]^8^)	Becker et al. 1997																			
29	Tv-29-ll-IVe (positional isomer of 4)	Mukherjee et al. 2011																			
30	Gelatinosin-B 8 (cf. hypomurocin B-4: [Vxx]^8^→[Lxx]^8^)	Becker et al. 1997																			
31	Gelatinosin-B 9 (cf. hypomurocin B-3b: [Vxx]^8^→[Lxx]^8^, [Vxxol]^18^ → [Lxxol]^l8^)	Becker et al. 1997																			
19	Gelatinosin-B 1 (cf. hypomurocin B-5: [Vxx]^8^→[Lxx]^8^)	Becker et al. 1997																			
32	Gelatinosin-B 10 (cf. 25: [Gln]^17^→ [Glu]^17^)																				
33	See *H. thelephoricola* (positional isomer of 5)																				
20	Gelatinosin-B 2 (cf. hypomurocin B-4: [Aib]^7^→[Vxx]^7^, [Vxx]^8^→[Lxx]^8^)	Becker et al. 1997																			
34	Gelatinosin-B 11 (cf. trichovirin II 6a and neoatroviridin C: [Gly]^2^→ [Ser]^2^)	Jaworski et al. 1999; Oh et al. 2005																			
6	See *H. thelephoricola*																				
25	Gelatinosin-B 5																				
27	Gelatinosin-B 6																				

a Variable residues are underlined in the table header. Minor sequence variants are underlined in the sequences. This applies to all sequence tables

**Table 8:** Sequences of 18- and 19-residue peptaibiotics detected in the specimen of *Hypocrea voglmayrii*

No.	t_R_ [min]	[*M*+ H]^+^		Residue^a^																		
				1	2	3	4	5	6	7	8	9	10	11	12	13	14	15	16	17	18	19
35	30.2–31.1	1762.0125	Ac	Aib	Ala	Aib	Ala	Aib	Gln	Aib	Aib	Aib	Ala	Lxx	Vxx	Pro	Vxx	Aib	Vxx	Glu	Gln	
36	31.6–32.0	1775.0433	Ac	Aib	Ala	Aib	Aib	Aib	Gln	Aib	Aib	Aib	Ala	Lxx	Vxx	Pro	Vxx	Aib	Vxx	Gln	Gln	
37	33.6–33.7	1924.1239	Ac	Aib	Ala	Aib	Aib	Aib	Gln	Aib	Aib	Aib	Ala	Lxx	Vxx	Pro	Vxx	Aib	Vxx	Gln	Gln	Tyrol
38	34.1–34.5	1911.1015	Ac	Aib	Ala	Ala	Aib	Aib	Gln	Aib	Aib	Aib	Ala	Lxx	Vxx	Pro	Vxx	Aib	Vxx	Gln	Glu	Tyrol
39	34.5–34.8	1925.1100	Ac	Aib	Ala	Aib	Aib	Aib	Gln	Aib	Aib	Aib	Ala	Lxx	Vxx	Pro	Vxx	Aib	Vxx	Gln	Glu	Tyrol
40	37.3–37.4	1880.1041	Ac	Aib	Ala	Ala	Aib	Aib	Gln	Aib	Aib	Aib	Ala	Lxx	Aib	Pro	Vxx	Aib	Vxx	Gln	Gln	Pheol
41	37.7–37.9	1894.1197	Ac	Aib	Ala	Aib	Aib	Aib	Gln	Aib	Aib	Aib	Ala	Lxx	Aib	Pro	Vxx	Aib	Vxx	Gln	Gln	Pheol
42	38.5–38.7	1881.0933	Ac	Aib	Ala	Ala	Aib	Aib	Gln	Aib	Aib	Aib	Ala	Lxx	Aib	Pro	Vxx	Aib	Vxx	Gln	Glu	Pheol
43	39.5–39.7	1894.1218	Ac	Aib	Ala	Ala	Aib	Aib	Gln	Aib	Aib	Aib	Ala	Lxx	Vxx	Pro	Vxx	Aib	Vxx	Gln	Gln	Pheol
44	39.9–40.1	1908.1391	Ac	Aib	Ala	Aib	Aib	Aib	Gln	Aib	Aib	Aib	Ala	Lxx	Vxx	Pro	Vxx	Aib	Vxx	Gln	Gln	Pheol
45	41.4–41.5	1909.1203	Ac	Aib	Ala	Aib	Aib	Aib	Gln	Aib	Aib	Aib	Ala	Lxx	Vxx	Pro	Vxx	Aib	Vxx	Gln	Glu	Pheol
46	42.8–43.0	1978.1743	Ac	Vxx	Ala	Ala	Aib	Aib	Gln	Aib	Aib	Aib	Ala	Lxx	Vxx	Pro	Vxx	Aib	Aib	Gln	Gln	Tyr(C_5_H_g_)ol^b^
47	43.4–43.6	1978.1741	Ac	Aib	Ala	Ala	Aib	Aib	Gln	Aib	Aib	Aib	Ala	Lxx	Vxx	Pro	Vxx	Aib	Vxx	Gln	Gln	Tyr(C_5_H_g_)ol
48	43.8–44.0	1992.1924	Ac	Aib	Ala	Aib	Aib	Aib	Gln	Aib	Aib	Aib	Ala	Lxx	Vxx	Pro	Vxx	Aib	Vxx	Gln	Gln	Tyr(C_5_H_g_)ol
49	44.6-44.7	1979.1585	Ac	Aib	Ala	Ala	Aib	Aib	Gln	Aib	Aib	Aib	Ala	Lxx	Vxx	Pro	Vxx	Aib	Vxx	Gln	Glu	Tyr(C_5_H_g_)ol
50	45.0–45.1	1993.1762	Ac	Aib	Ala	Aib	Aib	Aib	Gln	Aib	Aib	Aib	Ala	Lxx	Vxx	Pro	Vxx	Aib	Vxx	Gln	Glu	Tyr(C_5_H_g_)ol
51	45.9–46.1	2007.1881	Ac	Vxx	Ala	Aib	Aib	Aib	Gln	Aib	Aib	Aib	Ala	Lxx	Vxx	Pro	Vxx	Aib	Vxx	Gln	Glu	Tyr(C_5_H_g_)ol

No.	Compound identical or positionally isomeric with	Ref.																				
35	Voghiiayrin-1 (*N*-tenuinal heptapeptide, pos. 13–15 and 18 cf. trichokonin V)	Huang et al. 1995																				
36	Voglmayrin-2 (cf. 35: [Ala]^4^ → [Aib]^4^, [Glu]^17^→ [Gin]^17^: deletion sequence of 37)																					
37	Voglmayrin-3 (cf. 36: + *C*-tenninal Tyrol)																					
38	Voglmayrin-4																					
39	Voglmayrin-5 (cf. 37: [Gln]^18^→[Glu]^18^)																					
40	Voglmayrin-6 (*N*-tenninal nonapeptide cf. trichorzianine B-VIb, [Ser]^10^→[Ala]^10^, *C*-terminal nonapeptide cf. trichorzianine B-VIb, [Ile]^16^→ [Vxx]^16^)	Rebuffat et al. 1989																				
41	Voglmayrin-7																					
42	Voglmayrin-8 (homologue of 40: [Gln]^18^→[Glu]^18^)																					
43	Voglmayrin-9 (homologue of 40: [Aib]^12^ → [Vxx]^12^)																					
44	Voglmayrin-10 (homologue of 37: [Tyrol]^19^→ [Pheol]^19^)																					
45	Voglmayrin-11 (homologue of 39: [Tyrol] ^19^→[Pheol]^19^)																					
46	Voglmayrin-12																					
47	Voglmayrin-13 (homologue of 48: [Aib]^3^→ [Ala]^3^)																					
48	Voglmayrin-14 (homologue of 37 and 44: prenylated [Tyrol]^19^)																					
49	Voglmayrin-15 (homologue of 38: prenylated [Tyrol]^19^)																					
50	Voglmayrin-16 (homologue of 49: [Ala]^3^ →[Aib]^3^)																					
51	Voglmayrin-17 (homologue of 50: [Aib]^1^→[Vxx]^1^)																					

a Variable residues are underlined in the table header. Minor sequence variants are underlined in the sequences. This applies to all sequence tables

b C_5_H_8_, prenyl (Prn) or isoprenyl residue at OH-group of Tyr postulated. For details, see text

**Table 9:** Sequences of 11- and 19-residue peptaibiotics detected in the plate culture of *Hypocma voglmayrii*

No.	t_R_ [min]	[*M*+ H]^+^		Residue^a^																		
				1	2	3	4	5	6	7	8	9	10	11	12	13	14	15	16	17	18	19
52	35.2–35.6	1852.0739	Ac	Aib	Ala	Ala	Aib	Aib	Gln	Ala	Aib	Aib	Ala	Lxx	Aib	Pro	Vxx	Aib	Aib	Gln	Gln	Pheol
53	35.6–35.8	1866.0884	Ac	Aib	Ala	Ala	Aib	Aib	Gln	Ala	Aib	Aib	Ala	Lxx	Aib	Pro	Vxx	Aib	Vxx	Gln	Gln	Pheol
40	37.3–37.6	1880.1099	Ac	Aib	Ala	Ala	Aib	Aib	Gln	Aib	Aib	Aib	Ala	Lxx	Aib	Pro	Vxx	Aib	Vxx	Gln	Gln	Pheol
41	37.7–37.8	1894.1237	Ac	Aib	Ala	Aib	Aib	Aib	Gln	Aib	Aib	Aib	Ala	Lxx	Aib	Pro	Vxx	Aib	Vxx	Gln	Gln	Pheol
43	39.6–39.7	1894.1238	Ac	Aib	Ala	Ala	Aib	Aib	Gln	Aib	Aib	Aib	Ala	Lxx	Vxx	Pro	Vxx	Aib	Vxx	Gln	Gln	Pheol
44	40.0	1908.1395	Ac	Aib	Ala	Aib	Aib	Aib	Gln	Aib	Aib	Aib	Ala	Lxx	Vxx	Pro	Vxx	Aib	Vxx	Gln	Gln	Pheol
54	40.7–41.0	1052.7130	Oc	Aib	Gly	Lxx	Aib	Gly	Gly	Vxx	Aib	Gly	Lxx	Lxxol								
55	42.8–43.1	1066.7288	Oc	Aib	Gly	Lxx	Aib	Gly	Gly	Lxx	Aib	Gly	Lxx	Lxxol								

No.	Comment (compound identical or positionally isomeric with)	Ref.																				
52	Voglmayrin-18 (homologue of 53: [Vxx]^16^→[Aib]^16^; *N*-terminal hexapeptide cf. trichorzianine B-VIb; *C*-terminal nonapeptide cf. trichosporins B)	Rebuffat et al. 1989 Iida et al. 1990																				
53	Voglmayrin-19 (homologue of 40: [Aib]^7^→ [Ala]^7^; *C*-terminal nonapeptide cf. polysporin D)	New et al. 1996																				
40	Voglmayrin-20																					
41	Voglmayrin-21																					
43	Voglmayrin-22																					
44	Voglmayrin-23																					
54	cf. lipostrigocins B-04 and B-05	Degenkolb et al. 2006a																				
55	cf. trichogin A-IV	Auvin-Guette et al. 1992;																				
		Degenkolb et al. 2006a																				

a Variable residues are underlined in the table header. Minor sequence variants are underlined in the sequences. This applies to all sequence tables

**Table 10:** Sequences of 19-residue peptaibiotics detected in the specimen of *Hypocma minutispora*

No.	t_R_ [min]	[*M*+H]^+^		Residue^a^																		
				1	2	3	4	5	6	7	8	9	10	11	12	13	14	15	16	17	18	19
56	34.5–34.7	1847.1051	Ac	Aib	Ala	Aib	Gly	Aib	Gln	Aib	Lxx	Aib	Gly	Lxx	Aib	Pro	Vxx	Aib	Vxx	Glu	Gln	Lxxol
57	37.5–38.1	1846.1192	Ac	Aib	Ala	Aib	Ala	Aib	Gln	Aib	Lxx	Aib	Gly	Lxx	Aib	Pro	Vxx	Aib	Aib	Gln	Gln	Lxxol
58	38.5–38.6	1846.1099	Ac	Aib	Ala	Ala	Ala	Aib	Gln	Aib	Lxx	Aib	Gly	Lxx	Aib	Pro	Vxx	Aib	Vxx	Gln	Gln	Lxxol
59	39.1–39.4	1860.1278	Ac	Aib	Ala	Aib	Ala	Aib	Gln	Aib	Lxx	Aib	Gly	Lxx	Aib	Pro	Vxx	Aib	Vxx	Gln	Gln	Lxxol
60	39.8–40.1	1861.1130	Ac	Aib	Ala	Aib	Ala	Aib	Gln	Aib	Lxx	Aib	Gly	Lxx	Aib	Pro	Vxx	Aib	Vxx	Glu	Gln	Lxxol
61	40.9–41.0	1874.1420	Ac	Aib	Ala	Aib	Ala	Vxx	Gln	Aib	Lxx	Aib	Gly	Lxx	Aib	Pro	Vxx	Aib	Vxx	Gln	Gln	Lxxol
62	41.5–41.6	1875.1390	Ac	Aib	Ala	Aib	Aib	Aib	Gln	Aib	Lxx	Aib	Gly	Lxx	Aib	Pro	Vxx	Aib	Vxx	Glu	Gln	Lxxol
63	41.9–42.0	1875.1284	Ac	Aib	Ala	Aib	Ala	Aib	Gln	Aib	Lxx	Aib	Gly	Lxx	Aib	Pro	Lxx	Aib	Vxx	Glu	Gln	Lxxol

No.	Compound identical or positionally isomeric with	Ref.																				
56	Minutisporin-1 (pos. 1–3, 6, 7, 11–16, 18 and 19: cf. trichostrigocins A and B)	Degenkolb et al. 2006a																				
57	Minutisporin-2 (cf. hypophellin-18: [Pheol] 19→ [Lxxol]19; pos 1, 6, 7, 9, and the *C*-terminal nonapeptide: tricholongin B-I)	Rebuffat et al. 1991																				
58	Minutisporin-3 (cf. hypophellin-19: [Pheol]^19^ → [Lxxol]^19^; trichosporin B-IIIb – [Aib]^6^, [Pheol]^19^→ [Lxxol]^19^)	Röhrich et al. 2013a, b; Iida et al. 1990																				
59	Minutisporin-4 (cf. hypophellin-20: [Pheol]^19^→ [Lxxol]^19^; cf. trichosporin B-VIa – [Aib]^6^, [Aib]1^6^ → [Vxx]^16^, [Pheol]^19^ → [Lxxol]^19^; *C*-terminal nonapeptide, cf. tricholongin B-II; cf. trichocellin A-5 – [Ala]^6^, [Pheol]^20^→[Lxxol]^20^)	Rebuffat et al. 1991; Wada et al. 1994																				
60	Minutisporin-5 (*C*-terminal octapeptide, cf. hypelcin B-III)	Matsuura et al. 1994																				
61	Minutisporin-6 (cf. hypophellin-22: [Pheol]^19^→ [Lxxol]^19^; trichorzin HA-V: [Vxx]^5^–[Pro]^13^ and *C*-terminus with [Lxx]^14^ → [Vxx]^14^)	Hlimi et al. 1995; Röhrich et al. 2013a																				
62	Minutisporin-7																					
63	Minutisporin-8																					

a Variable residues are underlined in the table header. Minor sequence variants are underlined in the sequences. This applies to all sequence tables

**Table 11:** Sequences of 19-residue peptaibiotics detected in the plate culture of *Hypocrea minutispora*

No.	t_R_ [min]	[*M*+ H]^+^		Residue^a^																		
				1	2	3	4	5	6	7	8	9	10	11	12	13	14	15	16	17	18	19
64	36.1–36.3	1832.1060	Ac	Aib	Ala	Aib	Ala	Aib	Gln	Aib	Lxx	Aib	Gly	Lxx	Aib	Pro	Vxx	Aib	Aib	Gln	Gln	Vxxol
65	37.3–37.5	1832.1025	Ac	Aib	Ala	Aib	Gly	Aib	Gln	Aib	Lxx	Aib	Gly	Lxx	Aib	Pro	Vxx	Aib	Vxx	Gln	Gln	Vxxol
66	37.5–37.9	1846.1196	Ac	Aib	Ala	Aib	Ala	Aib	Gln	Aib	Lxx	Aib	Gly	Vxx	Aib	Pro	Vxx	Aib	Vxx	Gln	Gln	Lxxol
57	37.8–38.0	1846.1199	Ac	Aib	Ala	Aib	Ala	Aib	Gln	Aib	Lxx	Aib	Gly	Lxx	Aib	Pro	Vxx	Aib	Aib	Gln	Gln	Lxxol
67	38.6–38.7	1847.1135	Ac	Aib	Ala	Aib	Ala	Aib	Gln	Aib	Lxx	Aib	Gly	Lxx	Aib	Pro	Vxx	Aib	Aib	Glu	Gln	Lxxol
59	39.0–39.2	1860.1318	Ac	Aib	Ala	Aib	Ala	Aib	Gln	Aib	Lxx	Aib	Gly	Lxx	Aib	Pro	Vxx	Aib	Vxx	Gln	Gln	Lxxol
60	39.8–40.0	1861.1271	Ac	Aib	Ala	Aib	Ala	Aib	Gln	Aib	Lxx	Aib	Gly	Lxx	Aib	Pro	Vxx	Aib	Vxx	Glu	Gln	Lxxol
68	40.4–40.6	1874.1492	Ac	Aib	Ala	Aib	Ala	Aib	Gln	Aib	Lxx	Aib	Gly	Lxx	Aib	Pro	Lxx	Aib	Vxx	Gln	Gln	Lxxol
61	40.6–40.9	1874.1554	Ac	Aib	Ala	Aib	Ala	Vxx	Gln	Aib	Lxx	Aib	Gly	Lxx	Aib	Pro	Vxx	Aib	Vxx	Gln	Gln	Lxxol

No.	Compound identical or positionally isomeric with	Ref.																				
64	Minutisporin-9 (pos. 1, 6–10, 12–19; [Pro]^2^→ [Ala]^2^, [Aib]^11^→[Lxx]^11^ and deletion of [Aib]^5^: cf. stilboflavin B-5)	Jaworski and Brückner 2001b																				
65	Minutisporin-10 (positional isomer of 64: [Ala]^4^→[Gly]^4^, [Aib]^16^→[Vxx]^16^)																					
66	Minutisporin-11 (positional isomer of 57: [Lxx]^11^→[Vxx]^11^, [Aib]^16^→[Vxx]^16^)																					
57	Minutisporin-2																					
67	Minutisporin-12 (positional isomer of 57: [Gln]^17^→[Glu]^17^ and of 56: [Ala]^4^→[Gly]^4^, [Aib]^16^→[Vxx]^16^)																					
59	Minutisporin-4																					
60	Minutisporin-5																					
68	Minutisporin-13 (positional isomer of 61: [Aib]^5^→[Vxx]^5^)																					
61	Minutisporin-6																					

a Variable residues are underlined in the table header. Minor sequence variants are underlined in the sequences. This applies to all sequence tables

**Table 12:** Sequences of 19-residue peptaibiotics detected in the specimen of *Hypocrea citrina*

No.	t_R_ [min]	[*M*+H]^+^		Residue^a^																		
				1	2	3	4	5	6	7	8	9	10	11	12	13	14	15	16	17	18	19
69	31.6–31.7	1926.1036	Ac	Aib	Ala	Aib	Ala	Aib	Gln	Aib	Lxx	Aib	Gly	Lxx	Aib	Pro	Vxx	Aib	Vxx	Gln	Gln	di-OH-Pheol
70	32.0–32.1	1896.0937	Ac	Aib	Ala	Aib	Ala	Aib	Gln	Aib	Lxx	Aib	Gly	Lxx	Aib	Pro	Vxx	Aib	Aib	Gln	Gln	Tyrol
71	32.9–33.1	1910.1084	Ac	Aib	Ala	Aib	Ala	Aib	Gln	Aib	Lxx	Aib	Gly	Lxx	Aib	Pro	Vxx	Aib	Vxx	Gln	Gln	Tyrol
72	33.6–33.9	1880.0971	Ac	Aib	Ala	Aib	Gly	Aib	Gln	Aib	Lxx	Aib	Gly	Lxx	Aib	Pro	Vxx	Aib	Vxx	Gln	Gln	Pheol
73	34.6–34.7	1880.0975	Ac	Aib	Ala	Ala	Ala	Aib	Gln	Aib	Lxx	Aib	Gly	Lxx	Aib	Pro	Vxx	Aib	Vxx	Gln	Gln	Pheol
74	36.4–36.6	1880.0999	Ac	Aib	Ala	Ala	Ala	Aib	Gln	Aib	Lxx	Aib	Gly	Lxx	Aib	Pro	Vxx	Aib	Vxx	Gln	Gln	Pheol
75	37.7–37.9	1880.1050	Ac	Aib	Ala	Aib	Ala	Aib	Gln	Aib	Lxx	Aib	Gly	Lxx	Aib	Pro	Vxx	Aib	Aib	Gln	Gln	Pheol
76	38.2–38.4	1880.1018	Ac	Aib	Ala	Ala	Ala	Aib	Gln	Aib	Lxx	Aib	Gly	Lxx	Aib	Pro	Vxx	Aib	Vxx	Gln	Gln	Pheol
77	38.8–39.1	1894.1241	Ac	Aib	Ala	Aib	Ala	Aib	Gln	Aib	Lxx	Aib	Gly	Lxx	Aib	Pro	Vxx	Aib	Vxx	Gln	Gln	Pheol
78	39.7–39.9	1895.1083	Ac	Aib	Ala	Aib	Ala	Aib	Gln	Aib	Lxx	Aib	Gly	Lxx	Aib	Pro	Vxx	Aib	Vxx	Glu	Gln	Pheol

No.	Compound identical or positionally isomeric with	Ref.																				
69	Hypocitrin-1 (homologue of hypophellin-15: [Tyrol]^19^→ [di-OH-Pheol]^19^)	Röhrich et al. 2013a																				
70	Hypocitrin-2 (homologue of hypophellin-15: [Vxx]^17^→[Aib]^17^)	Röhrich et al. 2013a																				
71	Hypophellin-15	Röhrich et al. 2013a																				
72	Hypocitrin-3 (positional isomer of 73, 74, and 76: [Ala]^3^→ [Aib]^3^, [Ala]^4^→[Gly]^4^)																					
73	Hypocitrin-4 (positional isomer of 75 and 77, homologue of hypophellin-17: [Vxx]^17^→[Aib]^17^)	Röhrich et al. 2013a																				
74	Hypocitrin-5 (positional isomer of 73 and 77, homologue of hypophellin-17: [Vxx]^17^→[Aib]^17^)	Röhrich et al. 2013a																				
75	Hypophellin-18	Röhrich et al. 2013a																				
76	Hypocitrin-6 (positional isomer of 73 and 75, homologue of hypophellin-17: [Vxx]^17^→[Aib]^17^)	Röhrich et al. 2013a																				
77	Hypophellin-20	Röhrich et al. 2013a																				
78	Hypocitrin-7 (homologue of 77: [Gin]^17^→[Glu]^17^)																					

a Variable residues are underlined in the table header. Minor sequence variants are underlined in the sequences. This applies to all sequence tables

**Table 13:** General building scheme of the sequences of *Hypoerea/Trichoderma* SFl-peptaibiotics screened (Röhrich et al. 2012, 2013a, this study)

	Residue																			
	1	2	3	4	5	6	7	8	9	10	11	12	13	14	15	16	17	18	19^a^	20^b^
Ac	Aib	Ala	Aib	Ala	Aib	Ala	Gln	Aib	Lxx	Aib	Gly	Lxx	Aib	Pro	Vxx	Aib	Vxx	Gln	Gln	Pheol
	(Vxx)	(Ser)	Ala	Aib	(Vxx)	(Aib)		(Vxx)	Aib	(Ala)	Ala	(Vxx)	(Vxx)		Lxx	(Vxx)	Aib	(Glu)	-	Lxxol
		(Aib)	(Ser)	(Lxx)	(Phe)			(Ala)	(Vxx)		(Ser)	(Aib)			(Aib)		(Lxx)		(Glu)	(Vxxol)
		(Lxx)	(Vxx)	(Ser)	(Ala)															(Tyrol)
		(Vxx)		(Gly)	(Lxx)															(Tyr(C_5_H_8_)ol)
																				(di-OH-Pheol)

Minor sequence variants are parenthesised

a One of the Gln/Glu residues is deleted in some of the truncated sequences

b The *C*-terminal amino alcohol is deleted in some of the truncated sequences

**Table 14:** Phylogenetically verified peptaibiotic-producing strains and species of *Trichodermal Hypocrea* NB: Species and strains for which only MALDI-TOF-MS screening data have been published are not considered for inclusion

Species	Positively screened strains	Peptaibiotics found	References
*T. arundinaceum*	CBS 119575 (ex-type)	alamethicins F30	Degenkolb et al. 2008
		alamethicins F50	
		trichobrevins A	
		trichobrevins B	
		trichocompactins	
		trichoferin A	
	CBS 119576 (= **ATCC 90237**)^a^	trichobrevins A	Degenkolb et al. 2006b
		trichobrevins B	
		alamethicins F30	
		trichocompactins	
		trichoferins	
		trichocryptins B	
	CBS 119577	trichobrevins A	
		alamethicins F30	
		trichobrevins B	
		trichocompactins	
		trichoferin A	
	CBS 121153	alamethicins F30	Degenkolb et al. 2008
		alamethicins F50	
		trichobrevins A	
		trichobrevins B	
		trichocompactins	
		trichoferin A	
	CBS 123793 (= **NRRL 3199**)	alamethicins F30	Kirschbaum et al. 2003; Psurek et al. 2006; Degenkolb et al. 2006b, Degenkolb et al. 2008
		alamethicins F50	
		trichobrevins A	
		trichobrevins B	
		trichocompactins	
		trichoferins	
*T. brevicompactum*	CBS 109720 (= DAOM 231232, ex-type)	alamethicins F30	Degenkolb et al. 2006b
		trichocryptins A	
		trichocryptins B	
		trichocompactins	
	CBS 112444	alamethicins F30	Degenkolb et al. 2008
		trichocompactins	
		trichocryptins A	
		trichocryptins B	
		trichoferin A	
	CBS 112446	alamethicins F30	
	CBS 112447	alamethicins F50	
		trichocompactins	
		trichocryptins A	
		trichocryptins B	
		trichoferins	
	CBS 119569	alamethicins F30	Degenkolb et al. 2006b
	CBS 119570	trichocryptins A	
		trichocompactins	
*T. turrialbense*	CBS 112445 (ex-type)	alamethicins F30	Degenkolb et al. 2006b; Degenkolb et al. 2008
		trichocryptins A	
		trichocryptins B	
		trichocompactins	
	CBS 122554	alamethicins F30	Degenkolb et al. 2008
		alamethicins F50	
		trichocryptins C	
		trichocryptins D	
		trichocompactins	
		trichoferin A	
		(trichobrevins A)	
		(trichobrevins B)	
*T. protrudens*	CBS 121320 (ex-type)	trichobrevins A	Degenkolb et al. 2008
		trichobrevins B	
		alamethicins F30	
		alamethicins F50	
		trichocompactins	
		trichoferins	
*T. strigosum*	CBS 348.93 (ex-type)	tricholongins	Degenkolb et al. 2006a
		trichobrevins	
		trichostrigocins	
		trikoningins	
		trichogin AIV	
*T*. cf. *strigosum*	CBS 119777	tricholongins	
		lipostrigocins A	
		lipostrigocins B	
*T. erinaceus*	CBS 117088 (= DAOM 230019, ex-type)	trichostrigocins	
		trikoningin KB II	
*T. pubescens*	CBS 345.93 (= DAOM 166162, ex-type)	tricholongins	
		lipostrigocins	
*T*. cf. *pubescens*	CBS 119776	lipopubescin	
*T. stromaticum*	CBS 101875 (holotype)	trichostromaticins	
		trichocompactins	
	CBS 101730		
*T. spirale*	CBS 346.93 (ex-type)	trichobrevins B	
*H. rodmanii*	CBS 109719	hypocompactins	Degenkolb et al. 2008
	CBS 120897	hyporodicins	
		trichokonins	
*T. asperellum*	CBS 361.97^b^ (ATCC 38501, **NRRL 5242**)	trichotoxins A-50	Przybylski et al. 1984
		trichotoxins A-40	Jaworski and Bruckner 1999
	CBS 433.97 (ex-type)	trichotoxins A-50	Krause et al. 2006
	T32	trichotoxins	Chutrakul et al. 2008
	Y19-07	asperelines	Ren et al. 2009; 2013; Chen et al. 2013
*T. harzianum*	CBS 354.33 (= **CECT 2413** = ATCC 48131)	11-, 14-, and 18-residue peptaibols (not sequenced)	Vizcaíno et al. 2006
*T*. cf. *harzianum*	CBS 130670^c^ (ATCC 90200, NRRL 5243)	trichovirins II	Jaworski et al. 1999
*T. virens*	Tv29-8	trichorzins (18-residue peptaibols), 11- and 14-residue peptaibols	Wiest et al. 2002 Mukherjee et al. 2011
*T. polysporum*	**TMI60146**	trichopolyns	Fuji et al. 1978; Fujita et al. 1981; Iida et al. 1999
		trichosporins-B	Fujita et al. 1988, Iida et al. 1990; Iida et al. 1993
	**FKI-4452**	trichosporins-B	Iwatsuki et al. 2010
*T. reesei* (*H.jecorina*)	CBS 392.92 (ATCC 2692, **QM 9414**)	paracelsins	Brückner and Graf 1983; Brückner et al. 1984
*T. parareesei*	C.P.K. 618	hypojecorins-A	Degenkolb et al. 2012
	C.P.K. 665	hypojecorins-B	
		paracelsins	
*T. saturnisporum*	CBS 330.70 (ex-type)	paracelsin E	Ritieni et al. 1995
*T. atroviride*	**IFO 31288^d^**	hypomurocins A	Becker et al. 1997
		hypomurocins B	
	CBS 391.92^e^ (= **ATCC 36042**)	trichorzianins	El Hajji et al. 1987
	ATCC 74058^f^ (= PI) and mutants thereof	trichorzianins, trichoatrokontins	Pócsfalvi et al. 1998; Stoppacher et al. 2007, 2008
	MMS 639	unprecedented 17-residue peptaibiotics and 19-residue peptaibols	Carroux et al. 2013
	MMS 925		
	MMS 927		
	MMS 1295		
	MMS 1513		
*T. atroviride*	NF16	new and recurrent trichorzianins	Panizel et al. 2013
*T. citrinoviride*	**IMI 91968** ^g^	trichoaureocins	Jaworski and Bruckner 2001a
	**S25**	20-residue peptaibols	Maddau et al. 2009
*T. longibrachiatum*	DAOM 234100 (= MMS 151) Thb Thd CNM-CM 2171 (= C.P.K. 1696) CNM-CM 2277 (= C.P.K. 2277) IMI291014 (= C.P.K. 1303) CECT 2412 (= C.P.K. 2062) CECT 20105 (= C.P.K. 1698 = IMI 297702)	11-residue trichobrachins^h^ 11- and 20-residue trilongins	Mohamed-Benkada et al. 2006; Ruiz et al. 2007 Mikkola et al. 2012
*T. ghanense (syn. T. parceramosum)*	CBS 936.69^i^	trichobrachins	Brückner et al. 1993; Krause et al. 2007
*H. pulvinata*	CBS 133228	hypopulvins	Röhrich et al. 2012
	CBS 133229		
	CBS 133230		
*H. phellinicola* (ex-type)	CBS 119283	hypophellins	Röhrich et al. 2013
*H. peltata*	Not deposited	hypelcins	Fujita et al. 1984; Matsuura et al. 1993, 1994
*T. deliquescens (= G. deliquescens = G. viride*)^j^	CBS 228.48 (= ATCC 10097)	gliodeliquescin A	Brückner and Przybylski 1984
*T. flavofuscum (ex-type; syn. T. viiens*: Chaverri and Samuels [2003])	CBS 248.59 (= ATCC 13398 = DSM 3500 = IMI100714)	trichofumins	Berg et al. 2003

*T. asperellum*	CBS 433.97	only partial sequences were given, for comments on sequencing/putative identification of peptaibiotics, see Krause et al. (2006) 7	
*T. aggressivum var. europaeum*	CBS 100526		
*T. inhamatum*	CBS 345.96		
*H. dichromospora*	CBS 337.69		
*H. vinosa*	CBS 247.63		
*H. semiorbis*	CBS 244.63		
*H. citrina (syn. H. lactea)*	CBS 853.70		
*H. nigricans*	MUCL 28439		

*H. lactea*	IFO 8434	screened positive for peptidic Aib and Iva	Brückner et al. 1991
*H. schweinitzii*	ICMP 5421	screened positive for peptidic Aib	

a Accession numbers under which the peptaibiotic-producing strain was first published are highlighted in bold.

b Originally misidentified as *T. viride* (Hou et al. 1972).

c Originally misidentified as *T. viride* (Hou et al. 1972).

d Originally misidentified as *H. muroiana*, for taxonomic revision see Samuels et al. (2006).

e Originally misidentified as *T. harzianum* (el Hajji et al. 1987), for reidentificatian see Kuhls et al. (1996).

f Originally misidentified as *T. harzianum*.

g Originally misidentified as *T. aureoviride*; data taken from http://www.herbimi.info/herbimi/specimen.htm?imi=91968

h Not identical to those trichobrachins reported by Brückner et al. (1993) and Krause et al. (2007) from *T. ghanense* CBS 936.69.

i Originally misidentified as *T. longibrachiatum*.

j For taxonomic recombination of *G. deliquescens*, the anamorph of *H. lutea*, see Jaklitsch (2011).

## Footnotes

1 Authors are aware of the drastic change of the ICBN (International Code of Botanical Nomenclature), which has been adopted at the IBC in Melbourne in July 2011 (Gams et al. 2012; Rossman et al. 2013). However, all strains used in this study were deposited at CBS in July/August 2012, and practical work for this study was finished in December 2012. For reasons of conformity with recently published contributions in the field of peptaibiotics, dual nomenclature is retained in this chemically focussed article.

2 The trichorzianin-producing strain ATCC 36042 (= CBS 391.92) has originally been identified as *T. harzianum* (el Hajji et al. 1987) but later shown to belong to *T. atroviride* (Kuhls et al. 1996).

3 Neither a specimen, nor a culture of the hypelcin producer has been deposited. However, misidentification of *H. peltata* is impossible due to its cushion-like big stromata and distinctive bicellular ascospores (Samuels and Ismaiel 2011).

4 Defined as the dynamic entirety of peptaibiotics formed by a producing fungus under defined culture conditions (Krause et al. 2006a).

5 The trichotoxin A-producing strain NRRL 5242 (now A-18169 in the ARS culture collection=CBS 361.97=ATCC 38501) has originally been identified as *T. viride* but was subsequently reidentified as *T. asperellum* (Lieckfeldt et al. 1999; Samuels et al. 1999). The trichotoxin B (= trichovirin) producer, strain NRRL 5243 (= ATCC 90200), is not in the ARS catalogue but available as A-18207.

6 Hypomurocins have been isolated from strain IFO 31288 (Becker et al. 1997), originally misidentified as *Hypocrea muroiana*. The producer belongs, in fact, to *T. atroviride* (Samuels et al. 2006).

7 The neoatroviridin producer *T. atroviride* F80317 (Oh et al. 2005) has neither been deposited with an IDA, nor has its identity been verified phylogenetically.

8 Nielsen KF, Samuels GJ (2013) unpublished results.

9 Trichokonin VI is identical to gliodeliquescin A that has been isolated from *Gliocladium deliquescens* NRRL 1086 (Brückner et al. 1988) and not from NRRL 3091 (Brückner and Przybylski 1984). According to phylogenetic data, *G. deliquescens* NRRL 1086 (= CBS 228.48=ATCC 10097) was re-identified as *G. viride*, see (www.straininfo.net/strains/260309).
