# Supplementary material for: Front line defenders of the ecological niche! Screening the structural diversity of peptaibiotics from saprotrophic and fungicolous Trichoderma/Hypocrea species
Source: Fungal Divers. Author manuscript; Available in PMC 2015 Feb 24. (PMC4338523; doi:10.1007/s13225-013-0276-z)
Supplement: Supplementary Tables 1-9 [file NIHMS60077-supplement-suppl.doc]

**Table S1** Diagnostic fragment ions of 11- and 18-residue peptaibiotics detected in the specimen of *Hypocrea* *thelephoricola*

| Diagnostic fragment ions [*m/z*] | | Compound | | | | | | | | | |
| --- | --- | --- | --- | --- | --- | --- | --- | --- | --- | --- | --- |
|  |  | **1** | **2** | **3** | **4** | **5** | **6** | **7** | **8** | **9** | **10** |
| t_R_ [min] |  | 37.6-37.9 | 37.6-37.9 | 39.3-39.5 | 39.3-40.0 | 41.5-41.7 | 42.7-43.0 | 44.2-44.5 | 44.8-45.0 | 45.2-46.0 | 47.5-47.8 |
| [*M* + Na]^+^ |  | 1183.7345 | 1183.7345 | 1197.7468 | 1197.7468 | 1211.7626 | 1225.7793 | n.d. | 1768.0599 | n.d. | 1796.0904 |
| [*M* + H]^+^ |  | 1161.7527 | 1161.7527 | 1175.7712 | 1175.7712 | 1189.7836 | 1203.7981 | 1732.0673 | 1746.0866 | 1760.1035 | 1774.1161 |
| [*M* + H-H_2_O]^+^ |  | n.d.[[1]](#footnote-2) | n.d. | n.d. | 1157.7568 | n.d. | n.d. | n.d. | 1728.0719 | n.d. | n.d. |
| *a_1_* |  | n.d. | n.d. | n.d. | n.d. | n.d. | 114.0955 | n.d. | n.d. | n.d. | n.d. |
| *a_3_* |  | 228.1357 | 228.1340 | 228.1357 | 228.1345 | 228.1365 | 242.1520 | n.d. | n.d. | n.d. | n.d. |
| *a_4_* |  | 327.2035 | 327.2023 | 327.2035 | 341.2185 | 341.2192 | 355.2328 | n.d. | 256.1644 | 270.1788 | 270.1821 |
| *a_5_* |  | 440.2871 | 426.2710 | 440.2871 | 454.2934 | 454.3025 | 468.3148 | n.d. | 327.2005 | 341.2205 | 341.2229 |
| *b_1_* |  | 128.0711 | 128.0711 | 128.0726 | 128.0724 | 128.0724 | 142.0874 | 128.0732 | 128.0711 | 128.0706 | 128.0701 |
| *b_2_* |  | 256.1304 | 256.1295 | 256.1304 | 256.1303 | 256.1303 | 270.1447 | 199.1137 | 199.1091 | 199.1150 | 199.1118 |
| *b_3_* |  | 355.1981 | 355.1969 | 355.1981 | 369.2138 | 369.2138 | 383.2279 | 284.1627 | 284.1607 | 298.1763 | 298.1751 |
| *b_4_* |  | 468.2823 | 454.2670 | 468.2823 | 482.2958 | 482.2973 | 496.3135 | 355.1990 | 355.1977 | 369.2136 | 369.2118 |
| *b_5_* |  | 553.3367 | 539.3187 | 553.3366 | 567.3496 | 567.3510 | 581.3658 | 454.2654 | 454.2651 | 468.2813 | 468.2808 |
| *b_6_* |  | 650.3918 | 636.3710 | 650.3855 | 664.4010 | 664.4018 | 678.4171 | 582.3274 | 582.3263 | 596.3388 | 596.3388 |
| *b_7_* |  | 749.4535 | 749.4535 | 763.4723 | 763.4723 | 777.4849 | 791.4990 | 667.3775 | 667.3782 | 681.3932 | 681.3932 |
| *b_8_* |  | 862.5377 | 862.5377 | 876.5539 | 876.5539 | 890.5680 | 904.5827 | 766.4409 | 780.4610 | 794.4752 | 794.4752 |
| *b_9_* |  | 947.5931 | 947.5931 | 961.6057 | 961.6057 | 975.6210 | 989.6366 | 851.4940 | 865.5125 | 879.5273 | 879.5273 |
| *b_10_* |  | n.d. | n.d. | 1058.6442 | 1058.6442 | n.d. | 1086.6848 | 908.5195 | 922.5339 | 936.5480 | 936.5480 |
| *b_11_* |  |  |  |  |  |  |  | 1021.5980 | 1035.6150 | 1049.6329 | 1049.6319 |
| *b_12_* |  |  |  |  |  |  |  | 1106.6521 | 1120.6698 | 1134.6863 | 1134.6837 |
| *b_13_* |  |  |  |  |  |  |  | 1203.7140 | 1217.7306 | 1231.7423 | 1231.7527 |
| *b_14_* |  |  |  |  |  |  |  | 1316.7995 | n.d. | 1344.8132 | 1344.8376 |
| *b_15_* |  |  |  |  |  |  |  | 1401.8372 | 1415.8589 | 1429.8731 | 1429.8785 |
| *b_16_* |  |  |  |  |  |  |  | 1500.9088 | 1514.9247 | 1528.9447 | 1528.9396 |
| *b_17_* |  |  |  |  |  |  |  | 1628.9642 | 1642.9803 | 1657.0014 | 1656.9975 |

**Table S2** Diagnostic fragment ions of 11- and 18-residue peptaibiotics detected in the plate culture of *Hypocrea* *thelephoricola*

| Diagnostic fragment  ions [*m/z*] | | Compound | | | | | | | | | |
| --- | --- | --- | --- | --- | --- | --- | --- | --- | --- | --- | --- |
|  |  | **11** | | **1** | **2** | **12** | **5** | **13** | **6** | **8** | **9** |
| t_R_ [min] |  | | 35.6-35.8 | 37.2-37.4 | 37.7-37.9 | 39.5-40.0 | 40.6-40.8 | 41.5-41.7 | 42.8-43.0 | 44.8-44.9 | 45.5-45.7 |
| [*M* + Na]^+^ |  | | 1169.7259 | 1183.7443 | 1183.7441 | 1197.7613 | 1211.7773 | 1211.7774 | 1225.7935 | 1768.0767 | 1782.0888 |
| [*M* + H]^+^ |  | | 1147.7443 | 1161.7623 | 1161.7652 | 1175.7747 | 1189.7893 | 1189.7996 | 1203.8004 | 1746.0955 | 1760.1104 |
| [*M* + H-H_2_O]^+^ |  | | n.d. | 1143.7412 | n.d. | 1157.7561 | n.d. | n.d. | n.d. | n.d. | n.d. |
| *a_1_* |  | | n.d. | n.d. | n.d. | n.d. | n.d. | n.d. | 114.0915 | n.d. | n.d. |
| *a_2_* |  | | 228.1343 | 228.1341 | 228.1417 | 228.1353 | 228.1357 | 242.1475 | 242.1512 | 256.1645 | 270.1799 |
| *a_3_* |  | | 327.2011 | 327.2031 | 327.2035 | 341.2198 | 341.2196 | 341.2196 | 355.2355 | 327.2036 | 341.2236 |
| *a_4_* |  | | 426.2717 | 440.2853 | 426.2731 | 440.2886 | 454.3041 | 454.3041 | 468.3191 | n.d. | n.d. |
| *b_1_* |  | | 128.0703 | 128.0706 | 128.0705 | 128.0717 | 128.0718 | 142.0871 | 142.0876 | 128.0711 | 128.0712 |
| *b_2_* |  | | 256.1300 | 256.1301 | 256.1308 | 256.1317 | 256.1324 | 270.1469 | 270.1472 | 199.1140 | 199.1185 |
| *b_3_* |  | | 355.1989 | 355.1986 | 355.1993 | 369.2160 | 369.2122 | 369.2118 | 383.2316 | 284.1617 | 298.1771 |
| *b_4_* |  | | 454.2681 | 468.2810 | 454.2651 | 468.2815 | 482.2960 | 482.2953 | 496.3165 | 355.1968 | 369.2126 |
| *b_5_* |  | | 539.3192 | 553.3351 | 539.3199 | 553.3366 | 567.3509 | 567.3508 | 581.3703 | 454.2567 | 468.2806 |
| *b_6_* |  | | 636.3730 | 650.3872 | 636.3711 | 650.3870 | 664.4017 | 664.4030 | 678.4178 | 582.3265 | 596.3423 |
| *b_7_* |  | | 735.4392 | 749.4546 | 749.4559 | 763.4726 | 777.4854 | 777.4847 | 791.5026 | 667.3799 | 681.3959 |
| *b_8_* |  | | 848.5242 | 862.5382 | 862.5386 | 876.5559 | 890.5678 | 890.5669 | 904.5861 | 780.4614 | 794.4794 |
| *b_9_* |  | | 933.5770 | 947.5912 | 947.5925 | 961.6099 | 975.6219 | 975.6225 | 989.6411 | 865.5143 | 879.5340 |
| *b_10_* |  | | 1030.6254 | n.d. | n.d. | n.d. | n.d. | n.d. | n.d. | 922.5345 | 936.5535 |
| *b_11_* |  | |  |  |  |  |  |  |  | 1035.6181 | 1049.6374 |
| *b_12_* |  | |  |  |  |  |  |  |  | 1120.6692 | 1134.6899 |
| *y_6_* |  | |  |  |  |  |  |  |  | 626.4229 | 626.4228 |
| *y_6_* – H_2_O |  | |  |  |  |  |  |  |  | 608.4127 | 608.4125 |
| *y_6_* – AA (18) |  | |  |  |  |  |  |  |  | 523.3223 | 523.3213 |
| *y_6_* – AA (18-17) | |  | |  |  |  |  |  |  | 395.2636 | 395.2637 |
| *y_6_* – AA (18-16) | |  | |  |  |  |  |  |  | 296.1976 | 296.1973 |

**Table S3** Diagnostic fragment ions of 11- and 18-residue peptaibiotics detected in the specimen of *Hypocrea gelatinosa*

| Diagnostic fragment  ions [*m/z*] | | Compound | | | | | | | | | |
| --- | --- | --- | --- | --- | --- | --- | --- | --- | --- | --- | --- |
|  |  | **14** | | **15** | **16** | **17** | **18** | **19** | **20** | **21** | **22** |
| t_R_ [min] |  | | 37.1-37.3 | 37.7-37.8 | 38.0-38.2 | 38.8-38.9 | 39.5-39.6 | 40.2-40.4 | 40.9-41.1 | 41.2-41.4 | 41.9 |
| [*M* + Na]^+^ |  | | 1888.0712 | 1917.0875 | 1930.1163 | 1931.0988 | 1902.0855 | 1784.0669 | 1784.0641 | 1798.0813 | 1974.1456 |
| [*M* + H]^+^ |  | | 1866.0929 | 1895.1067 | 1908.1358 | 1909.1186 | 1880.1083 | 1762.0856 | 1762.0840 | 1776.1023 | 1952.1674 |
| *a_1_* |  | | 100.0768 | 100.0773 | 100.0787 | 100.0811 | 100.0769 | 100.0798 | 100.0796 | 100.0790 | n.d. |
| *a_2_* |  | | n.d. | 171.1215 | n.d. | n.d. | n.d. | n.d. | n.d. | 187.1234 | n.d. |
| *a_3_* |  | | 242.1544 | 256.1697 | 256.1697 | 256.1676 | 242.1536 | 258.1461 | 258.1481 | 258.1485 | n.d. |
| *b_1_* |  | | 128.0740 | 128.0749 | 128.0748 | 128.0751 | 128.0752 | 128.0755 | 128.0749 | 128.0755 | n.d. |
| *b_2_* |  | | 199.1143 | 199.1139 | 199.1137 | 199.1144 | 199.1153 | 215.1090 | 215.1091 | 215.1089 | n.d. |
| *b_2_* – H_2_O |  | | n.d. | n.d. | n.d. | n.d. | n.d. | n.d. | 197.0985 | 197.0984 | n.d. |
| *b_3_* |  | | 270.1505 | 284.1652 | 284.1652 | 284.1641 | 270.1521 | 286.1448 | 286.1438 | 286.1365 | n.d. |
| *b_3_* – H_2_O |  | | n.d. | n.d. | n.d. | n.d. | n.d. | 268.1348 | 268.1350 | 268.1352 | n.d. |
| *b_4_* |  | | 341.1863 | 369.2183 | 369.2160 | 369.2179 | 341.1843 | 399.2277 | 399.2238 | 399.2242 | 369.2123 |
| *b_5_* |  | | 426.2383 | 516.2878 | 516.2874 | 516.2873 | 426.2383 | 484.2801 | 484.2814 | 498.2948 | 516.2775 |
| *b_6_* |  | | 554.3033 | 644.3361 | 644.3567 | 644.3327 | 554.2962 | 612.3417 | 612.3409 | 626.3567 | 644.3403 |
| *b_7_* |  | | 639.3512 | 729.3997 | 729.3989 | 729.3987 | 639.3529 | 697.3936 | 711.4100 | 711.4086 | 729.3956 |
| *b_8_* |  | | 752.4359 | 814.4508 | 814.4519 | 814.4445 | 752.4397 | 810.4779 | 824.4946 | 824.4925 | 814.4478 |
| *b_9_* |  | | 837.4862 | 899.5030 | 899.4921 | 899.4937 | 837.4908 | 895.5327 | 909.4583 | 909.5449 | 899.5020 |
| *b_10_* |  | | 894.5093 | 956.5168 | 956.5104 | 956.5073 | 908.5291 | 952.5524 | 966.5649 | 966.5654 | 986.5384 |
| *b_10_* – H_2_O |  | | n.d. | n.d. | n.d. | n.d. | n.d. | n.d. | n.d. | n.d. | 968.5275 |
| *b_11_* |  | | 1007.5953 | 1069.6001 | 1069.6069 | 1069.5922 | 1021.6089 | 1051.6193 | 1065.6320 | 1065.6366 | 1099.6062 |
| *b_12_* |  | | 1092.6452 | 1154.6630 | 1154.6630 | 1154.6630 | 1106.6557 | 1136.6736 | 1150.6889 | 1150.6867 | 1184.6717 |
| *y_6_* |  | |  |  |  |  |  | 626.4321 | 612.3862 | 626.4219 |  |
| *y_6_* – H_2_O |  | |  |  |  |  |  | n.d. | n.d. | 608.4091 |  |
| *y_6_* – AA (18) |  | |  |  |  |  |  | 509.3031 | 509.3097 | 509.3143 |  |
| *y_6_* – AA (18-17) | |  | |  |  |  |  | 381.2508 | 381.2534 | 381.2464 |  |
| *y_6_* – AA (18-16) | |  | |  |  |  |  | 296.1992 | 296.2024 | 296.1983 |  |
| *y_7_* |  | | 774.4661 | 741.4580 | 754.4870 | 755.4834 | 774.4569 |  |  |  | 768.4989 |
| *y_7_* – H_2_O |  | | n.d. | 723.4497 | n.d. | n.d. | n.d. |  |  |  | 750.4859 |
| *y_7_* – AA (19) |  | | 623.3547 | 624.3404 | 637.3792 | 638.3568 | 623.3446 |  |  |  | 651.3785 |
| *y_7_* – AA (19-18) | | 495.3009 | | 496.2785 | 509.3125 | 510.2982 | 495.2881 |  |  |  | 523.3188 |
| *y_7_* – AA (19-17) | | 367.2369 | | 367.2404 | 381.2507 | 381.2564 | 367.2425 |  |  |  | 395.2613 |
| y*_7_* – AA (19-16) | | 282.1574 | | 282.1840 | n.d. | n.d. | n.d. |  |  |  | 310.2187 |

**Table S3** (continued)

| Diagnostic fragment  ions [*m/z*] | | Compound | | | | | | | | | | | |
| --- | --- | --- | --- | --- | --- | --- | --- | --- | --- | --- | --- | --- | --- |
|  |  | **23** | | | **6** | | **24** | | **25** | | **26** | | **27** |
| t_R_ [min] |  | | 42.1-42.3 | 42.3 | | 42.9 | | 43.0-43.1 | | 44.6 | | 45.8 | |
| [*M* + Na]^+^ |  | | 1798.0813 | 1225.7937 | | 1975.1322 | | n.d. | | 1941.1390 | | 1796.1125 | |
| [*M* + H]^+^ |  | | 1776.1023 | 1203.8117 | | 1953.1515 | | 1790.1199 | | 1919.1568 | | 1774.1299 | |
| *a_1_* |  | | 100.0790 | n.d. | | n.d. | | 100.0791 | | 100.0788 | | 100.0780 | |
| *a_2_* |  | | n.d. | n.d. | | n.d. | | n.d. | | n.d. | | 171.1388 | |
| *a_3_* |  | | 258.1492 | n.d. | | n.d. | | n.d. | | 256.1688 | | 242.1547 | |
| *b_1_* |  | | 128.0759 | 142.0913 | | n.d. | | 128.0761 | | 128.0760 | | 128.0757 | |
| *b_2_* |  | | 215.1097 | 270.1504 | | n.d. | | 215.1090 | | 199.1156 | | 199.1148 | |
| *b_2_* – H_2_O |  | | 197.0987 | n.d. | | n.d. | | 197.0987 | | n.d. | | n.d. | |
| *b_3_* |  | | 286.1508 | 383.2301 | | n.d. | | 286.1455 | | 284.1653 | | 270.1508 | |
| *b_3_* – H_2_O |  | | 268.1357 | n.d. | | n.d. | | 268.1358 | | n.d. | | n.d. | |
| *b_4_* |  | | 399.2226 | 496.3072 | | 369.2112 | | 399.2221 | | 369.2144 | | 383.2260 | |
| *b_5_* |  | | 498.2931 | 581.3628 | | 516.2775 | | 498.2916 | | 482.2945 | | 482.2954 | |
| *b_6_* |  | | 626.3537 | 678.4131 | | 644.3425 | | 626.3529 | | 610.3534 | | 610.3472 | |
| *b_7_* |  | | 725.4209 | 791.5048 | | 729.3942 | | 725.4214 | | 695.4054 | | 709.4237 | |
| *b_8_* |  | | 838.5048 | n.d. | | 814.4465 | | 838.5049 | | 780.4627 | | 822.5103 | |
| *b_9_* |  | | 923.5568 | n.d. | | 899.5003 | | 923.5565 | | 865.5168 | | 907.5668 | |
| *b_10_* |  | | 980.5821 | n.d. | | 986.5321 | | 980.5770 | | 952.5469 | | 964.6021 | |
| *b_10_* – H_2_O |  | | n.d. | n.d. | | 968.5217 | | n.d. | | 934.5452 | | n.d. | |
| *b_11_* |  | | 1079.6456 | 1203.8117 | | 1099.6228 | | 1079.6477 | | 1065.6366 | | 1063.6464 | |
| *b_12_* |  | | 1164.7070 |  | | 1184.6667 | | 1164.6967 | | 1150.6914 | | 1148.7097 | |
| *y_6_* |  | | 612.4081 |  | |  | | 626.4224 | |  | | 626.4213 | |
| *y_6_* – H_2_O |  | | 594.3985 |  | |  | | 608.4123 | |  | | 608.4064 | |
| *y_6_* – AA (18) |  | | 509.3055 |  | |  | | 509.3030 | |  | | 509.3052 | |
| *y_6_* – AA (18-17) |  | | 381.2472 |  | |  | | 381.2462 | |  | | 381.2470 | |
| *y_6_* – AA (18-16) |  | | 296.1999 |  | |  | | 296.1983 | |  | | 296.1988 | |
| *y_7_* |  | |  |  | | 769.4834 | |  | |  | |  | |
| *y_7_* – H_2_O |  | |  |  | | 751.4721 | |  | |  | |  | |
| *y_7_* – AA (19) |  | |  |  | | 652.3642 | |  | |  | |  | |
| *y_7_* – AA (19-18) |  | |  |  | | 524.3070 | |  | |  | |  | |
| *y_7_* – AA (19-17) |  | |  |  | | 395.2640 | |  | |  | |  | |
| y*_7_* – AA (19-16) |  | |  |  | | 310.2187 | |  | |  | |  | |

**Table S4** Diagnostic fragment ions of 11- and 18-residue peptaibiotics detected in the plate culture of *Hypocrea gelatinosa*

| Diagnostic fragment ions [*m/z*] | Compound | | | | | | | |
| --- | --- | --- | --- | --- | --- | --- | --- | --- |
|  | **28** | **29** | **30** | **31** | **19** | **32** | **33** | **20** |
| t_R_ [min] | 38.0-38.1 | 38.8-38.9 | 39.2-39.3 | 39.4-39.7 | 40.1-40.4 | 40.5-40.7 | 40.8-41.0 | 40.9-41.1 |
| [*M* + Na]^+^ | 1770.0603 | 1197.7660 | 1770.0603 | n.d. | n.d. | n.d. | 1211.7824 | n.d. |
| [*M* + H]^+^ | 1748.0789 | 1175.7832 | 1748.0789 | 1762.0802 | 1762.0814 | 1777.0993 | 1189.8026 | 1762.0797 |
| [*M* + H-H_2_O]^+^ | n.d. | n.d. | n.d. | 1744.0609 | 1744.0623 | 1759.0822 | n.d. | 1744.0650 |
| *a_2_* | n.d. | 228.1419 | n.d. | n.d. | n.d. | n.d. | 228.1402 | n.d. |
| *a_3_* | n.d. | 341.2205 | n.d. | n.d. | n.d. | n.d. | 341.2237 | n.d. |
| *b_1_* | 128.0720 | 128.0723 | 128.0729 | 128.0730 | 128.0729 | 128.0729 | 128.0730 | 128.0729 |
| *b_2_* | 215.1081 | 256.1368 | 215.1092 | 215.1086 | 215.1089 | 215.1085 | 256.1362 | 215.1092 |
| *b_2_* – H_2_O | 197.0974 | n.d. | 197.0979 | 197.0979 | 197.0982 | 197.0979 | n.d. | 197.0981 |
| *b_3_* | 286.1452 | 369.2127 | 286.1458 | 286.1458 | 286.1454 | 286.1455 | 369.2142 | 286.1455 |
| *b_3_* – H_2_O | 268.1348 | n.d. | 268.1353 | 268.1356 | 268.1353 | 268.1355 | n.d. | 268.1352 |
| *b_4_* | 399.2282 | 482.2951 | 399.2292 | 399.2207 | 399.2234 | 399.2258 | 482.2975 | 399.2221 |
| *b_5_* | 484.2746 | 567.3490 | 484.2751 | 484.2764 | 484.2740 | 498.2924 | 567.3523 | 484.2768 |
| *b_6_* | 612.3373 | 664.3920 | 612.3375 | 612.3367 | 612.3362 | 626.3529 | 664.4052 | 612.3317 |
| *b_7_* | 697.3886 | 763.4726 | 697.3911 | 711.4053 | 697.3867 | 725.4227 | 777.4886 | 711.4043 |
| *b_8_* | 810.4664 | 876.5533 | 810.4740 | 824.4816 | 810.4793 | 838.5044 | 890.5732 | 824.4879 |
| *b_9_* | 895.5224 | 961.6068 | 895.5257 | 909.5376 | 895.5219 | 923.5540 | 975.6284 | 909.5392 |
| *b_10_* | 952.5413 |  | 952.4566 | 966.5618 | 952.5488 | 980.5730 |  | 966.5612 |
| *b_11_* | 1037.5991 |  | 1051.6162 | 1051.6116 | 1051.6127 | 1065.6327 |  | 1065.6255 |
| *b_12_* | 1122.6575 |  | 1136.6744 | 1136.6630 | 1136.6671 | 1150.6820 |  | 1150.6825 |
| *b_13_* | n.d. |  | n.d. | n.d. | 1233.7223 | 1247.7346 |  | 1247.7290 |
| *b_14_* | n.d. |  | n.d. | n.d. | 1346.8037 | n.d. |  | n.d. |
| *b_15_* | n.d. |  | n.d. | 1431.8475 | 1431.8478 | 1445.8652 |  | 1445.8672 |
| *b_16_* | n.d. |  | n.d. | 1516.9058 | 1516.9038 | 1530.9192 |  | 1530.9189 |
| *b_17_* | n.d. |  | n.d. | 1644.9647 | 1644.9542 | 1659.9785 |  | 1658.9830 |
| PLLU |  | 409.2819 |  |  |  |  | 409.2809 |  |
| *y_6_* | 626.4156 |  | 612.4114 | n.d. | n.d. | n.d. |  | n.d. |
| *y_6_* – H_2_O | 608.4109 |  | 594.3968 | n.d. | n.d. | n.d. |  | n.d. |
| *y_6_* – AA (18) | 509.3092 |  | 509.3072 | n.d. | n.d. | n.d. |  | n.d. |
| *y_6_* – AA (18-17) | 381.2494 |  | 381.2518 | n.d. | n.d. | n.d. |  | n.d. |
| *y_6_* – AA (18-16) | 296.1988 |  | 296.2023 | n.d. | n.d. | n.d. |  | n.d. |

**Table S4** (continued)

| Diagnostic fragment  ions [*m/z*] | Compound | | | |
| --- | --- | --- | --- | --- |
|  | **34** | **6** | **25** | **27** |
| t_R_ [min] | 41.8-42.1 | 42.7-42.9 | 43.1-43.3 | 45.7-46.0 |
| [*M* + Na]^+^ | n.d. | 1225.7997 | n.d. | 1796.1188 |
| [*M* + H]^+^ | 1776.1016 | 1203.8234 | 1790.1139 | 1774.1162 |
| [*M* + H-H_2_O]^+^ | 1758.0861 | n.d. | 1772.0969 | 1756.1035 |
| *a_2_* | n.d. | 242.1557 | n.d. | n.d. |
| *a_3_* | n.d. | 355.2394 | n.d. | n.d. |
| *b_1_* | 128.0730 | 142.0897 | 128.0731 | 128.0730 |
| *b_2_* | 215.1088 | 270.1515 | 215.1086 | 199.1147 |
| *b_2_* – H_2_O | 197.0981 | n.d. | 197.0981 | n.d. |
| *b_3_* | 286.1458 | 383.2350 | 286.1459 | 270.1507 |
| *b_3_* – H_2_O | 268.1357 | n.d. | 268.1359 | n.d. |
| *b_4_* | 399.2249 | 496.3044 | 399.2248 | 383.2342 |
| *b_5_* | 484.2759 | 581.3653 | 498.2929 | 482.3030 |
| *b_6_* | 612.3373 | 678.4204 | 626.3494 | 610.3627 |
| *b_7_* | 711.4068 | 791.5068 | 725.4187 | 709.4258 |
| *b_8_* | 824.4894 | 904.5920 | 838.5001 | 822.5068 |
| *b_9_* | 909.5422 | 989.6491 | 923.5558 | 907.5615 |
| *b_10_* | 966.5625 |  | 980.5733 | 964.5822 |
| *b_11_* | 1065.6338 |  | 1079.6443 | 1063.6487 |
| *b_12_* | 1150.6865 |  | 1164.6996 | 1148.7034 |
| *b_13_* | 1247.7426 |  | 1261.7575 | n.d. |
| *b_14_* | 1360.8155 |  | 1374.8346 | n.d. |
| *b_15_* | 1445.8755 |  | n.d. | 1443.9028 |
| *b_16_* | 1530.9235 |  | 1545.9422 | 1528.9470 |
| *b_17_* | 1658.9817 |  | 1672.9946 | 1657.0023 |
| PLLU |  | 409.2636 |  |  |
| *y_6_* | n.d. |  | n.d. | n.d. |
| *y_6_* – H_2_O | n.d. |  | n.d. | n.d. |
| *y_6_* – AA (18) | n.d. |  | n.d. | n.d. |
| *y_6_* – AA (18-17) | n.d. |  | n.d. | n.d. |
| *y_6_* – AA (18-16) | n.d. |  | n.d. | n.d. |

**Table S5** Diagnostic fragment ions of 11- and 19-residue peptaibiotics detected in the specimen of *Hypocrea voglmayrii*

| Diagnostic fragment ions [*m/z*] | Compound | | | | | | | | | |
| --- | --- | --- | --- | --- | --- | --- | --- | --- | --- | --- |
|  | **35** | **36** | **37** | **38** | **39** | **40** | **41** | **42** | **43** | **44** |
| t_R_ [min] | 30.2-31.1 | 31.6-32.0 | 33.6-33.7 | 34.1-34.5 | 34.5-34.8 | 37.3-37.4 | 37.7-37.9 | 38.5-38.7 | 39.5-39.7 | 39.9-40.1 |
| [*M* + Na]^+^ | n.d. | 1797.0040 | 1946.1115 | 1933.0791 | 1947.0922 | 1902.0837 | 1916.0982 | 1903.0735 | 1916.1055 | 1930.1191 |
| [*M* + H]^+^ | 1762.0125 | 1775.0433 | 1924.1239 | 1911.1015 | 1925.1100 | 1880.1041 | 1894.1197 | 1881.0933 | 1894.1218 | 1908.1391 |
| *a_1_* | n.d. | n.d. | n.d. | n.d. | n.d. | n.d. | n.d. | n.d. | n.d. | n.d. |
| *a_3_* | 256.1642 | 256.1653 | 256.1615 | 242.1512 | 256.1622 | 242.1515 | 256.1659 | 242.1524 | 242.1519 | 256.1674 |
| *a_4_* | 327.1980 | n.d. | 341.1835 | 327.2020 | 341.1853 | 327.2055 | n.d. | 327.2064 | 327.2052 | n.d. |
| *b_1_* | 128.0662 | 128.0679 | 128.0693 | 128.0697 | 128.0694 | 128.0721 | 128.0721 | 128.0722 | 128.0723 | 128.0725 |
| *b_2_* | 199.1115 | 199.1098 | 199.1098 | 199.1098 | 199.1104 | 199.1097 | 199.1100 | 199.1104 | 199.1102 | 199.1101 |
| *b_3_* | 284.1614 | 284.1620 | 284.1619 | 270.1472 | 284.1627 | 270.1477 | 284.1626 | 270.1486 | 270.1476 | 284.1634 |
| *b_4_* | 355.1991 | 369.2153 | 369.2137 | 355.1990 | 369.2147 | 355.1998 | 369.2109 | 355.2012 | 355.2004 | 369.2125 |
| *b_5_* | 440.2468 | 454.2671 | 454.2638 | 440.2479 | 454.2362 | 440.2519 | 454.2647 | 440.2567 | 440.2495 | 454.2600 |
| *b_6_* | 568.3079 | 582.3273 | 582.3275 | 568.3086 | 582.3260 | 568.3005 | 582.3253 | 568.3209 | 568.3083 | 582.3246 |
| *b_7_* | 653.3566 | 667.3783 | 667.3770 | 653.3585 | 667.3774 | 653.3644 | 667.3780 | 653.3682 | 653.3627 | 667.3787 |
| *b_8_* | 738.4070 | 752.4303 | 752.4294 | 738.4110 | 752.4284 | 738.4171 | 752.4287 | 738.4160 | 738.4166 | 752.4306 |
| *b_9_* | 823.4668 | 837.4801 | 837.4819 | 823.4651 | 837.4810 | 823.4697 | 837.4817 | 823.4659 | 823.4684 | 837.4839 |
| *b_10_* | 894.5007 | 908.5119 | 908.5140 | 894.5021 | 908.5177 | 894.5046 | 908.5170 | 894.5035 | 894.5045 | 908.5194 |
| *b_11_* | 1007.5841 | 1021.5981 | 1021.6024 | 1007.5824 | 1021.6019 | 1007.5871 | 1021.6024 | 1007.5860 | 1007.5882 | 1021.6031 |
| *b_12_* | 1106.6519 | 1120.6688 | 1120.6701 | 1106.6521 | 1120.6703 | 1092.6364 | 1106.6542 | 1092.6390 | 1106.6570 | 1120.6733 |
| y*_7_* + C_5_H_8_ | n.d. | n.d. | n.d. | n.d. | n.d. | n.d. | n.d. | n.d. | n.d. | n.d. |
| *y_7_* | n.d. | n.d. | 804.4582 | 805.4454 | 805.4437 | 788.4643 | 788.4635 | 789.4527 | 788.4650 | 788.4650 |
| *y_7_* – H_2_O | n.d. | n.d. | n.d. | n.d. | n.d. | 770.4491 | 770.4536 | n.d. | 770.4482 | 770.4506 |
| *y_7_* – AA (19) | n.d. | n.d. | 637.3636 | 638.3512 | 638.3470 | 637.3648 | 637.3660 | 638.3535 | 637.3647 | 637.3650 |
| *y_7_* – AA (19-18) | n.d. | n.d. | 509.3036 | 509.3055 | 509.3068 | 509.3032 | 509.3068 | 509.3062 | 509.3053 | 509.3048 |
| *y_7_* – AA (19-17) | n.d. | n.d. | 381.2448 | 381.2529 | 381.2547 | 381.2457 | 381.2488 | 381.2482 | 381.2472 | 381.2474 |
| y*_7_* – AA (19-16) | n.d. | n.d. | 282.1763 | 282.1763 | 282.1687 | 282.1705 | 282.1789 | n.d. | 282.1819 | 282.1819 |
| *y_6_* | 656.3650 | 655.3870 |  |  |  |  |  |  |  |  |
| *y_6_* – AA (18) | 510.2951 | 509.3227 |  |  |  |  |  |  |  |  |
| *y_6_* – AA (18-17) | 381.2472 | 381.2448 |  |  |  |  |  |  |  |  |
| y*_6_* – AA (18-16) | 282.1763 | n.d. |  |  |  |  |  |  |  |  |

**Table S5** (continued)

| Diagnostic fragment ions [*m/z*] | Compound | | | | | | |
| --- | --- | --- | --- | --- | --- | --- | --- |
|  | **45** | **46** | **47** | **48** | **49** | **50** | **51** |
| t_R_ [min] | 41.4-41.5 | 42.8-43.0 | 43.4-43.6 | 43.8-44.0 | 44.6-44.7 | 45.0-45.1 | 45.9-46.1 |
| [*M* + Na]^+^ | 1931.1045 | 2000.1622 | 2000.1562 | 2014.1707 | 2001.1448 | 2015.1581 | 2029.1791 |
| [*M* + H]^+^ | 1909.1203 | 1978.1743 | 1978.1741 | 1992.1924 | 1979.1585 | 1993.1762 | 2007.1881 |
| *a_1_* | n.d. | n.d. | n.d. | n.d. | n.d. | n.d. | 114.0920 |
| *a_3_* | n.d. | 256.1673 | 242.1517 | 256.1671 | 242.1518 | 256.1673 | 270.1747 |
| *a_4_* | n.d. | n.d. | 327.2048 | n.d. | 327.2056 | n.d. | n.d. |
| *b_1_* | 128.0722 | 142.0880 | 128.0720 | 128.0725 | 128.0720 | 128.0719 | 142.0880 |
| *b_2_* | 199.1102 | 213.1251 | 199.1097 | 199.1102 | 199.1099 | 199.1099 | 213.1254 |
| *b_3_* | 284.1638 | 284.1630 | 270.1475 | 284.1634 | 270.1470 | 284.1632 | 298.1774 |
| *b_4_* | 369.2122 | 369.2127 | 355.1986 | 369.2106 | 355.1998 | 369.2133 | 383.2276 |
| *b_5_* | 454.2640 | 454.2649 | 440.2481 | 454.2642 | 440.2491 | 454.2627 | 468.2793 |
| *b_6_* | 582.3250 | 582.3258 | 568.3062 | 582.3242 | 568.3084 | 582.3238 | 596.3411 |
| *b_7_* | 667.3777 | 667.3772 | 653.3613 | 667.3782 | 653.3627 | 667.3778 | 681.3943 |
| *b_8_* | 752.4292 | 752.4297 | 738.4144 | 752.4294 | 738.4143 | 752.4294 | 766.4438 |
| *b_9_* | 837.4811 | 837.4805 | 823.4662 | 837.4813 | 823.4666 | 837.4812 | 851.4982 |
| *b_10_* | 908.5170 | 908.5172 | 894.5024 | 908.5172 | 894.5025 | 908.5180 | 922.5337 |
| *b_11_* | 1021.6002 | 1021.6024 | 1007.5850 | 1021.6004 | 1007.5843 | 1021.6002 | 1035.6180 |
| *b_12_* | 1120.6705 | 1120.6686 | 1106.6528 | 1120.6702 | 1106.6529 | 1120.6693 | 1134.6869 |
| y*_7_* + C_5_H_8_ | n.d. | 858.5091 | 872.5249 | 872.5260 | 873.5088 | 873.5059 | 873.5057 |
| *y_7_* | 789.4482 | 790.4489 | 804.4614 | 804.4620 | 805.4499 | 805.4432 | 805.4411 |
| *y_7_* – H_2_O | n.d. | n.d. | n.d. | n.d. | n.d. | n.d. | n.d. |
| *y_7_* – AA (19) | 638.3518 | 623.3510 | 637.3681 | 637.3668 | 638.3531 | 638.3537 | 638.3488 |
| *y_7_* – AA (19-18) | 509.3071 | 495.2902 | 509.3079 | 509.3086 | 509.3079 | 509.3066 | 509.3062 |
| *y_7_* – AA (19-17) | 381.2476 | 367.2330 | 381.2495 | 381.2492 | 381.2526 | 381.2506 | 381.2478 |
| y*_7_* – AA (19-16) | 282.1870 | n.d. | 282.1865 | 282.1830 | 282.1782 | 282.1847 | 282.1738 |
| *y_6_* |  |  |  |  |  |  |  |
| *y_6_* – AA (18) |  |  |  |  |  |  |  |
| *y_6_* – AA (18-17) |  |  |  |  |  |  |  |
| y*_6_* – AA (18-16) |  |  |  |  |  |  |  |

**Table S6** Diagnostic fragment ions of 11-and 19-residue peptaibiotics detected in the plate culture of *Hypocrea voglmayrii*

| Diagnostic fragment ions [*m/z*] | Compound | | | | | | | |
| --- | --- | --- | --- | --- | --- | --- | --- | --- |
|  | **52** | **53** | **40** | **41** | **43** | **44** | **54** | **55** |
| t_R_ [min] | 35.2-35.6 | 35.6-35.8 | 37.3-37.6 | 37.7-37.8 | 39.6-39.7 | 40.0 | 40.7-41 | 42.8-43.1 |
| [*M* + Na]^+^ | 1874.0581 | 1888.0734 | 1902.0903 | 1916.1048 | 1916.1041 | 1930.1190 | 1074.6945 | 1088.7103 |
| [*M* + H]^+^ | 1852.0739 | 1866.0884 | 1880.1099 | 1894.1237 | 1894.1238 | 1908.1395 | 1052.7130 | 1066.7288 |
| *a_2_* | n.d. | n.d. | n.d. | n.d. | 171.1155 | 171.1152 | n.d. | n.d. |
| *a_3_* | 242.1556 | 242.1555 | 242.1519 | 242.1516 | 256.1671 | 256.1671 | 354.2759 | 354.2768 |
| *a_4_* | 327.2052 | 327.2055 | 327.2054 | 327.2040 | n.d. | n.d. | n.d. | n.d. |
| *b_1_* | 128.0817 | 128.0819 | 128.0715 | 128.0715 | 128.0715 | 128.0714 | 212.1659 | 212.1664 |
| *b_2_* | 199.1154 | 199.1155 | 199.1103 | 199.1101 | 199.1101 | 199.1099 | 269.1879 | 269.1879 |
| *b_3_* | 270.1502 | 270.1503 | 270.1503 | 270.1471 | 284.1590 | 284.1631 | 382.2713 | 382.2718 |
| *b_4_* | 355.1993 | 355.1986 | 355.1988 | 355.1964 | 369.2101 | 383.2254 | 467.3243 | 467.3247 |
| *b_5_* | 440.2497 | 440.2477 | 440.2502 | 440.2486 | 454.2646 | 468.2806 | 524.3464 | 524.3469 |
| *b_6_* | 568.3136 | 568.3134 | 568.3123 | 568.3077 | 582.3227 | 596.3427 | 581.3683 | 581.3689 |
| *b_7_* | 639.3519 | 639.3519 | 653.3636 | 653.3619 | 667.3778 | 681.3922 | 680.4361 | 694.4510 |
| *b_8_* | 724.4039 | 724.4030 | 738.4167 | 738.4141 | 752.4303 | 766.4453 | 765.4924 | 779.5024 |
| *b_9_* | 809.4555 | 809.4524 | 823.4693 | 823.4671 | 837.4826 | 851.4968 | n.d. | n.d. |
| *b_10_* | 880.4943 | 880.4926 | 894.5061 | 894.5039 | 908.5190 | 922.5343 | n.d. | n.d. |
| *b_11_* | 993.5773 | 993.5711 | 1007.5905 | 1007.5888 | 1021.6028 | 1035.6207 |  |  |
| *b_12_* | 1078.6316 | 1078.6256 | 1092.6455 | 1106.6564 | 1106.6557 | 1134.6872 |  |  |
| *y_7_* | 774.4515 | 788.4669 | 788.4666 | 788.4675 | 788.4669 | 788.4678 |  |  |
| *y_7_* – H_2_O | 757.4258 | 770.4521 | 770.4577 | 770.4553 | 770.4553 | 770.4580 |  |  |
| *y_7_* – AA (19) | 623.3552 | 637.3738 | 637.3641 | 637.3642 | 637.3621 | 637.3639 |  |  |
| *y_7_* – AA (19-18) | 495.2952 | 509.3041 | 509.3064 | 509.3069 | 509.3063 | 509.3072 |  |  |
| *y_7_* – AA (19-17) | 367.2340 | 381.2516 | 381.2492 | 381.2492 | 381.2485 | 381.2490 |  |  |
| y*_7_* – AA (19-16) | 282.1795 | 282.1793 | 282.1791 | 282.1819 | 282.1751 | 282.1817 |  |  |

**Table S7** Diagnostic fragment ions of 19-residue peptaibiotics detected in the specimen of *Hypocrea minutispora*

| Diagnostic fragment ions [*m/z*] | Compound | | | | | | | |
| --- | --- | --- | --- | --- | --- | --- | --- | --- |
|  | **56** | **57** | **58** | **59** | **60** | **61** | **62** | **63** |
| t_R_ [min] | 34.5-34.7 | 37.5-38.1 | 38.5-38.6 | 39.1-39.4 | 39.8-40.1 | 40.9-41.0 | 41.5-41.6 | 41.9-42.0 |
| [*M* + Na]^+^ | n.d. | 1868.0862 | n.d. | n.d. | 1883.0944 | n.d. | n.d. | n.d. |
| [*M* + H]^+^ | 1847.1051 | 1846.1192 | 1846.1099 | 1860.1278 | 1861.1130 | 1874.1420 | 1875.1390 | 1875.1284 |
| *a_1_* | 100.0777 | n.d. | 100.0795 | n.d. | 100.0792 | 100.0790 | 100.0797 | 100.0794 |
| *a_3_* | n.d. | 256.1691 | 242.1531 | 256.1701 | 256.1701 | 256.1695 | 256.1697 | 256.1695 |
| *a_4_* | n.d. | 327.2068 | 313.1888 | 327.2055 | 327.2056 | 327.2055 | 341.2211 | 327.2040 |
| *a_5_* | 256.1667 | 412.2570 | 398.2414 | 412.2572 | 412.2569 | 426.2636 | n.d. | 412.2561 |
| *b_1_* | 128.0753 | 128.0761 | 128.0758 | 128.0765 | 128.0761 | 128.0763 | 128.0756 | 128.0755 |
| *b_2_* | 199.1159 | 199.1149 | 199.1139 | 199.1163 | 199.1165 | 199.1166 | 199.1149 | 199.1154 |
| *b_3_* | 284.1641 | 284.1645 | 270.1488 | 284.1592 | 284.1658 | 284.1652 | 284.1650 | 284.1646 |
| *b_4_* | 341.1906 | 355.2005 | 341.1829 | 355.1965 | 355.1978 | 355.2008 | 369.2174 | 355.2009 |
| *b_5_* | 426.2334 | 440.2520 | 426.2341 | 440.2484 | 440.2508 | 454.2633 | 454.2693 | 440.2518 |
| *b_6_* | 554.2953 | 568.3122 | 554.2927 | 568.3100 | 568.3091 | 582.3233 | 582.3304 | 568.3150 |
| *b_7_* | 639.3486 | 653.3628 | 639.3460 | 653.3629 | 653.3631 | 667.3773 | 667.3824 | 653.3634 |
| *b_8_* | 752.4289 | 766.4457 | 752.4294 | 766.4468 | 766.4469 | 780.4607 | 780.4557 | n.d. |
| *b_9_* | 837.4826 | 851.4969 | 837.4822 | 851.4997 | 851.5004 | 865.5152 | 865.5107 | 851.4983 |
| *b_10_* | 894.5046 | 908.5148 | 894.5026 | 908.5206 | 908.5190 | 922.5389 | n.d. | n.d. |
| *b_11_* | 1007.5862 | n.d. | 1007.5900 | 1021.6013 | 1021.6041 | 1035.6185 | n.d. | n.d. |
| *b_12_* | 1092.6434 | 1106.6510 | 1092.6393 | 1106.6552 | 1106.6553 | 1120.6745 | 1120.6704 | 1106.6532 |
| *b_13_* | 1189.7164 | n.d. | n.d. | 1203.7081 | 1203.7142 | n.d. | n.d. | n.d. |
| *b_14_* | 1288.7853 | 1302.7745 | 1288.7653 | 1302.7718 | 1302.7707 | n.d. | n.d. | n.d. |
| *b_15_* | 1373.8288 | 1387.8531 | 1373.8141 | 1387.8248 | 1387.8251 | 1401.8604 | 1401.8604 | n.d. |
| *b_16_* | 1472.8849 | 1472.8896 | 1472.8900 | 1486.8953 | 1486.8932 | 1500.9020 | 1500.9020 | n.d. |
| *b_17_* | 1601.9265 | 1600.9553 | 1600.9439 | 1614.9524 | 1615.9394 | 1629.0123 | 1629.9398 | 1629.9726 |
| *b_18_* | 1729.9950 | 1729.0175 | 1729.0047 | 1743.0096 | 1743.9921 | 1757.0365 | 1758.0242 | 1758.0199 |

**Table S8** Diagnostic fragment ions of 19-residue peptaibiotics detected in the plate culture of *Hypocrea minutispora*

| Diagnostic fragment ions [*m/z*] | Compound | | | | | | | | |
| --- | --- | --- | --- | --- | --- | --- | --- | --- | --- |
|  | **64** | **65** | **66** | **57** | **67** | **59** | **60** | **68** | **61** |
| t_R_ [min] | 36.1-36.3 | 37.3-37.5 | 37.5-37.9 | 37.8-38.0 | 38.6-38.7 | 39.0-39.2 | 39.8-40.0 | 40.4-40.6 | 40.6-40.9 |
| [*M* + Na]^+^ | 1854.0864 | 1854.0873 | 1868.1014 | 1868.1011 | 1869.1011 | 1882.1185 | 1883.1093 | 1896.1314 | 1896.1446 |
| [*M* + H]^+^ | 1832.1060 | 1832.1025 | 1846.1196 | 1846.1199 | 1847.1135 | 1860.1318 | 1861.1271 | 1874.1492 | 1874.1554 |
| *a_3_* | 256.1646 | 256.1660 | 256.1661 | 256.1663 | 256.1658 | 256.1671 | 256.1647 | 256.1664 | 256.1650 |
| *a_4_* | 327.1948 | 313.1896 | 327.2050 | 327.2033 | 327.2029 | 327.2038 | 327.2043 | 327.2032 | 327.2028 |
| *a_5_* | 412.2491 | n.d. | 412.2560 | 412.2561 | 412.2581 | 421.2568 | 412.2570 | 412.2566 | n.d. |
| *b_1_* | 128.0706 | 128.0714 | 128.0715 | 128.0718 | 128.0717 | 128.0721 | 128.0718 | 128.0696 | 128.0710 |
| *b_2_* | 199.1117 | 199.1103 | 199.1112 | 199.1122 | 199.1108 | 199.1129 | 199.1124 | 199.1119 | 199.1127 |
| *b_3_* | 284.1616 | 284.1623 | 284.1622 | 284.1622 | 284.1625 | 284.1630 | 284.1624 | 284.1619 | 284.1621 |
| *b_4_* | 355.1954 | 341.1855 | 355.1957 | 355.1989 | 355.2014 | 355.1998 | 355.1911 | 355.1945 | 355.2030 |
| *b_5_* | 440.2457 | 426.2298 | 440.2506 | 440.2497 | 440.2553 | 440.2533 | 440.2492 | 440.2524 | 454.2672 |
| *b_6_* | 568.3074 | 554.2931 | 568.3092 | 568.3099 | 568.3163 | 568.3114 | 568.3095 | 568.3107 | 582.3337 |
| *b_7_* | 653.3660 | 639.3479 | 653.3659 | 653.3617 | 653.3651 | 653.3639 | 653.3656 | 653.3678 | 667.3849 |
| *b_8_* | 766.4463 | 752.4324 | 766.4458 | 766.4480 | 766.4483 | 766.4480 | 766.4486 | 766.4562 | 780.4612 |
| *b_9_* | 851.5022 | 837.4838 | 851.4998 | 851.5014 | 851.5008 | 851.5019 | 851.5013 | 851.4986 | 865.5135 |
| *b_10_* | 908.5165 | 894.5007 | 908.5182 | 908.5205 | 908.5259 | 908.5223 | 908.5212 | 908.5154 | 922.5463 |
| *b_11_* | 1021.6090 | 1007.5913 | 1007.5881 | 1021.6057 | 1021.6094 | 1027.6058 | 1021.6082 | 1021.6040 | 1035.6255 |
| *b_12_* | 1106.6594 | 1092.6419 | 1092.6429 | 1106.6632 | 1106.6600 | 1106.6578 | 1106.6592 | 1106.6574 | 1120.6743 |
| *y_7_* | 726.4533 | 740.4686 | 754.4833 | 740.4689 | 741.4529 | 754.4849 | 755.4690 | 768.4963 | 754.4844 |
| *y_7_* – H_2_O | 708.4432 | n.d. | 736.4606 | n.d. | 723.4433 | 736.4722 | 737.4664 | n.d. | 736.4834 |
| *y_7_* – AA (19) | 623.3500 | 637.3731 | 637.3635 | 623.3528 | 624.3318 | 637.3685 | 638.3521 | 651.3898 | 637.3738 |
| *y_7_* – AA (19-18) | 495.2915 | 509.3112 | 509.3037 | 495.2930 | 496.2762 | 509.3077 | 510.2921 | 523.3244 | 509.3013 |
| *y_7_* – AA (19-17) | 367.2348 | 381.2449 | 381.2449 | 367.2349 | 367.2361 | 381.2495 | 381.2501 | 395.2631 | 381.2473 |
| y*_7_* – AA (19-16) | 282.1828 | n.d. | n.d. | 282.1665 | 282.1775 | 282.1802 | 282.1839 | 296.2170 | n.d. |

**Table S9** Diagnostic fragment ions of 19-residue peptaibiotics detected in the specimen of *Hypocrea citrina*

| Diagnostic fragment ions [*m/z*] | Compound | | | | | | | | | |
| --- | --- | --- | --- | --- | --- | --- | --- | --- | --- | --- |
|  | **69** | **70** | **71** | **72** | **73** | **74** | **75** | **76** | **77** | **78** |
| t_R_ [min] | 31.6-31.7 | 32.0-32.1 | 32.9-33.1 | 33.6-33.9 | 34.6-34.7 | 36.4-36.6 | 37.7-37.9 | 38.2-38.4 | 38.8-39.1 | 39.7-39.9 |
| [*M* + Na]^+^ | 1948.0872 | 1918.0697 | 1932.0906 | 1902.0766 | 1902.0805 | 1902.0800 | 1902.0838 | 1902.0816 | 1916.1005 | 1917.0913 |
| [*M* + H]^+^ | 1926.1036 | 1896.0937 | 1910.1084 | 1880.0971 | 1880.0975 | 1880.0999 | 1880.1050 | 1880.1018 | 1894.1241 | 1895.1083 |
| *a_3_* | 256.2696 | 256.1701 | 256.1703 | 256.1697 | 242.1542 | 242.1544 | 256.1708 | 242.1544 | 256.1714 | 256.1711 |
| *a_4_* | 327.2023 | 327.2044 | 327.2058 | 313.1898 | 313.1909 | n.d. | 327.2070 | 313.1915 | 327.2077 | 327.2069 |
| *a_5_* | 412.2524 | 412.2564 | 412.2582 | n.d. | n.d. | 398.2425 | 412.2594 | 398.2456 | 412.2604 | 412.2597 |
| *b_1_* | 128.0728 | 128.0743 | 128.0753 | 128.0738 | 128.0729 | 128.0744 | 128.0757 | 128.0754 | 128.0762 | 128.0754 |
| *b_2_* | 199.1157 | 199.1154 | 199.1158 | 199.1161 | 199.1192 | 199.1199 | 199.1164 | 199.1147 | 199.1172 | 199.1165 |
| *b_3_* | 284.1659 | 284.1658 | 284.1663 | 284.1659 | 270.1497 | 270.1501 | 284.1667 | 270.1508 | 284.1677 | 284.1671 |
| *b_4_* | 355.1949 | 355.2022 | 355.2027 | 341.1883 | 341.1796 | 341.1884 | 355.2033 | 341.1890 | 355.2044 | 355.2038 |
| *b_5_* | 440.2468 | 440.2559 | 440.2559 | 426.2400 | 426.2332 | 426.2397 | 440.2564 | 426.2408 | 440.2574 | 440.2569 |
| *b_6_* | 568.3080 | 568.3073 | 568.3121 | 551.2954 | 554.2907 | 554.2970 | 568.3102 | 554.2927 | 568.3063 | 568.3064 |
| *b_7_* | 653.3595 | 653.3569 | 653.3636 | 639.3466 | 639.3451 | 639.3482 | 653.3632 | 639.3460 | 653.3599 | 653.3594 |
| *b_8_* | 766.4438 | 766.4436 | 766.4475 | 752.4313 | 752.4297 | 752.4315 | 766.4471 | 752.4300 | 766.4436 | 766.4430 |
| *b_9_* | 851.4961 | 851.4960 | 851.5011 | 837.4819 | 837.4822 | 837.4855 | 851.5008 | 837.4822 | 851.4962 | 851.4958 |
| *b_10_* | 908.5144 | 908.5189 | 908.5196 | 894.5028 | 894.4990 | 894.5062 | 908.5199 | 894.5016 | 908.5154 | 908.5149 |
| *b_11_* | 1021.6006 | 1021.5988 | 1021.6035 | 1007.5869 | 1007.5847 | 1007.5869 | 1021.6034 | 1007.5840 | 1021.5977 | 1021.5972 |
| *b_12_* | 1106.6508 | 1106.6496 | 1106.6583 | 1092.6385 | 1092.6370 | 1092.6404 | 1106.6580 | 1092.6370 | 1106.6497 | 1106.6485 |
| *b_12_* – H_2_O | n.d. | n.d. | 1088.6412 | n.d. | 1074.6247 | n.d. | 1088.6436 | 1074.6234 | 1088.6379 | 1088.6386 |
| *y_7_* | 820.4554 | 790.4446 | 804.4624 | 788.4669 | 788.4663 | 788.4674 | 774.4520 | 788.4642 | 788.4668 | 789.4519 |
| *y_7_* – H_2_O | n.d. | 772.4438 | n.d. | 770.4577 | n.d. | 770.4638 | 756.4410 | 770.4597 | 770.4517 | 771.4445 |
| *y_7_* – AA (19) | 637.3670 | 623.3490 | 637.3674 | 637.3660 | 637.3691 | 637.3664 | 623.3498 | 637.3650 | 637.3660 | 638.3502 |
| *y_7_* – AA (19-18) | 509.3067 | 495.2912 | 509.3074 | 509.3058 | 509.3037 | 509.3061 | 495.2919 | 509.3059 | 509.3068 | 510.2912 |
| *y_7_* – AA (19-17) | 381.2486 | 367.2354 | 381.2468 | 381.2495 | 381.2483 | 381.2517 | 367.2326 | 381.2492 | 381.2487 | 381.2487 |
| y*_7_* – AA (19-16) | 282.1783 | 282.2002 | 282.1834 | 282.1785 | n.d. | n.d. | 282.1834 | 282.1836 | 282.1857 | 282.1778 |

1. n. d., not detected. [↑](#footnote-ref-2)
